# Benchmarking differential expression analysis tools for RNA-Seq: normalization-based vs. log-ratio transformation-based methods

**DOI:** 10.1186/s12859-018-2261-8

**Published:** 2018-07-18

**Authors:** Thomas P. Quinn, Tamsyn M. Crowley, Mark F. Richardson

**Affiliations:** 1 Centre for Molecular and Medical Research, School of Medicine, Deakin University, Geelong, 3220 Australia; 2 Bioinformatics Core Research Group, Deakin University, Geelong, 3220 Australia; 3 Poultry Hub Australia, University of New England, Armidale, 2351 Australia; 4 Centre for Integrative Ecology, School of Life and Environmental Science, Deakin University, Geelong, 3220 Australia

**Keywords:** High-throughput sequencing analysis, RNA-Seq, Compositional data, Compositional analysis, CoDA

## Abstract

**Background:**

Count data generated by next-generation sequencing assays do not measure absolute transcript abundances. Instead, the data are constrained to an arbitrary “library size” by the sequencing depth of the assay, and typically must be normalized prior to statistical analysis. The constrained nature of these data means one could alternatively use a log-ratio transformation in lieu of normalization, as often done when testing for differential abundance (DA) of operational taxonomic units (OTUs) in 16S rRNA data. Therefore, we benchmark how well the ALDEx2 package, a transformation-based DA tool, detects differential expression in high-throughput RNA-sequencing data (RNA-Seq), compared to conventional RNA-Seq methods such as edgeR and DESeq2.

**Results:**

To evaluate the performance of log-ratio transformation-based tools, we apply the ALDEx2 package to two simulated, and two real, RNA-Seq data sets. One of the latter was previously used to benchmark dozens of conventional RNA-Seq differential expression methods, enabling us to directly compare transformation-based approaches. We show that ALDEx2, widely used in meta-genomics research, identifies differentially expressed genes (and transcripts) from RNA-Seq data with high precision and, given sufficient sample sizes, high recall too (regardless of the alignment and quantification procedure used). Although we show that the choice in log-ratio transformation can affect performance, ALDEx2 has high precision (i.e., few false positives) across all transformations. Finally, we present a novel, iterative log-ratio transformation (now implemented in ALDEx2) that further improves performance in simulations.

**Conclusions:**

Our results suggest that log-ratio transformation-based methods can work to measure differential expression from RNA-Seq data, provided that certain assumptions are met. Moreover, these methods have very high precision (i.e., few false positives) in simulations and perform well on real data too. With previously demonstrated applicability to 16S rRNA data, ALDEx2 can thus serve as a single tool for data from multiple sequencing modalities.

**Electronic supplementary material:**

The online version of this article (10.1186/s12859-018-2261-8) contains supplementary material, which is available to authorized users.

## Background

In the last decade, new technologies, collectively known as next generation sequencing (NGS), have come to dominate the market [1]. Although NGS has a wide range of applications, the use of NGS in transcriptome profiling, called massively parallel RNA-sequencing (RNA-Seq), is perhaps most popular [2]. Like microarray, RNA-Seq is used to quantify relative transcript abundance (i.e., expression) [1]. Unlike microarrays however, RNA-Seq is able to estimate the relative abundance of uncharacterized transcripts as well as differentiate between transcript isoforms [2]. Meanwhile, advances in NGS have reduced the cost of sequencing tremendously, making it possible to generate an enormous amount of raw sequencing data easily and cheaply.

However, the analysis of raw sequencing data is not trivial. The data, constituting a “library” of hundreds of thousands of short sequence fragments, must undergo a number of processing steps prior to relative abundance estimation [3]. In the setting of an established reference genome (or transcriptome), this process generally includes (1) removal of undesired sequences (e.g., assay-specific adapters, ribosomal RNA, or short reads) and quality filtering, (2) alignment of the remaining sequences to the reference, and (3) quantification of relative transcript abundance [3]. In addition, RNA-Seq has two notable sources of bias that an analyst may need to address: transcripts with longer lengths [4] and higher GC content [5] have their relative abundances over-estimated.

Alignment is a computationally expensive process that, in many cases, contributes to the major bottleneck in RNA-Seq workflows [6]. As the ability to generate raw sequence data appears to outpace gains in computing power, the advantage of fast alignment seems clear. Although dozens of aligners exist (e.g., see [7–10]), the STAR aligner [6] has grown in popularity as a method that balances accuracy with efficiency, having good performance in systematic evaluations [10, 11]. Recently, a new family of “pseudo-alignment” methods has emerged (e.g., Kallisto [12], Sailfish [13], and Salmon [14]), providing an order of magnitude faster speeds than conventional aligners [14]. On the other hand, quantification is comparatively quick and sometimes performed by the aligners themselves (as in the case of Salmon and STAR). In essence, quantification involves “counting” the number of times a sequence aligns to a given portion of the reference [3]. This results in a matrix of counts (or pseudo-counts) describing the estimated number of times each transcript was present for each sample under study (although some methods represent relative abundance in other units [15]). Yet, the choice in the alignment and quantification method used seems to matter less than the choice in the software used for down-stream analyses [16].

The “count matrix” produced by alignment and quantification is perhaps most commonly used for differential expression (DE) analysis, a means by which to identify which genes (or transcripts), if any, have a statistically significant difference in (ideally, absolute) abundance across the experimental groups [3]. Like alignment, dozens of methods exist for DE analysis, providing a unique approach to normalization and statistical modeling. Of these, DESeq [17] and edgeR [18] seem most popular. Both use a type of normalization whereby each “library” (i.e., sample vector) is adjusted by a scaling factor based on a reference (or pseudo-reference). DESeq uses as the reference the median of the ratios of each gene for that sample to the geometric mean of each gene for all samples [17, 19]. Meanwhile, edgeR uses as the reference the weighted mean of log ratios between that sample and an explicitly chosen reference, a method known as the trimmed mean of M (TMM) [19, 20]. Underlying this approach is the rarely stated assumption that most transcripts do not differ in relative abundance while gains and losses happen with equipoise [21].

While the choice in normalization can affect the final results of a DE analysis [22, 23], it is necessary because per-sample counts generated by alignment and quantification do not compare directly [24]. This is because a sequencer only sequences a fraction of the total input, thereby constraining the per-sample output to a fraction of the total number of molecules in the input library: the number of reads delivered, called the sequencing depth, is therefore a property of the sequencer itself (and not the sampled environment) [24]. As such, the increased presence of any one transcript in the input material results in a decreased measurement for all other transcripts [24]. This sum constraint makes RNA-Seq data a kind of compositional data in which each sample is a composition and the transcript-wise counts are the components [25]. Compositional data have two key properties. First, the total sum of all components is an artifact of the sampling procedure [26]. Second, the differences between any two components only carry meaning proportionally [26]. For example, the difference between the two counts [50, 500] is the same as the difference between [100, 1000] since the latter could be obtained from the former simply by doubling the sequencing depth. RNA-Seq data have both of these properties, but differ slightly from true compositional data in that count data only contain integer values [25, 27].

Compositional data analysis (CoDA) describes a collection of methods used to analyze compositional data, including those pioneered by Aitchison in 1986 [28]. Commonly, such analyses begin with a transformation, most often the centered log-ratio (clr) transformation (defined in Methods). In contrast to normalizations, these transformations do not claim to retrieve absolute abundances from the compositional data [29]. Yet, they are sometimes used as if they were normalizations themselves [29]. The ALDEx2 package (available for the R programming language) uses log-ratio transformations in lieu of normalization for the analysis of sequencing data [30]. This package, first developed to examine meta-genomics data [30, 31] (but subsequently shown to work for a broad range of high-throughput sequencing study designs [32]), identifies differentially abundant features across two or more groups by applying statistical hypothesis testing to compositional data in three steps: (1) generate Monte Carlo (MC) instances (of log-ratio transformed data) based on the provided count matrix using the Dirichlet distribution, (2) apply univariate statistical models on the MC instances, and (3) calculate the expected (i.e., average) adjusted false discovery rate (FDR) *p*-values per-transcript across all MC instances [30]. By default, ALDEx2 uses the clr transformation, although it supports other transformations too.

The ALDEx2 package is not well-adopted for RNA-Seq analysis, although its applicability to RNA-Seq is established elsewhere [32]. However, ALDEx2 is used to analyze 16S rRNA data (e.g., [33, 34]) where it is shown to achieve much lower false positive rates (FPR) than competing DE methods [35]. Yet, we do not know of any paper which independently benchmarks ALDEx2 as a DE method for RNA-Seq (excepting the aforementioned article written by the ALDEx2 authors [32]). Moreover, we do not know the extent to which the choice of log-ratio transformation influences the final results of an analysis. Finally, we do not know whether the results produced by ALDEx2 are sensitive to the chosen alignment and quantification method. In this paper, we use simulated and real data to evaluate the performance of ALDEx2 as a DE method for RNA-Seq data and demonstrate that ALDEx2, given a sufficient number of replicates, is appropriate for the analysis of RNA-Seq data. In doing so, we also present a novel log-ratio transformation, based on iterative runs of ALDEx2, that may improve accuracy when compared with other approaches.

## Methods

### Data acquisition

To benchmark how well the ALDEx2 package (available for the R programming language) performs as a differential expression method for RNA-Seq data, we analyzed four data sets. The first two contain simulated data generated from the polyester package (available for the R programming language) [36]. polyester simulates RNA-Seq data as raw sequencing data (i.e., FASTQ read sets) where the relative abundances of the transcripts follow a negative binomial model [36]. The third data set contained real data sourced from a previously published benchmark study [16], retrieved as raw RNA sequencing data (i.e., FASTQ read sets) from the NCBI SRA, under accession SRP082682 https://www.ncbi.nlm.nih.gov/Traces/study/?acc=srp082682. The fourth data set serves as an “everyday use case” example and comprises publicly available RNA-Seq data from a previous study on the adaptation and evolution of the invasive cane toad (*Rhinella marina*) [37]. The data set consists of 20 samples, 10 each from two experimental groups, made available from the NCBI SRA, under accession PRJNA277985 https://www.ncbi.nlm.nih.gov/bioproject/PRJNA419245. We used these raw data for sequencing alignment, quantification, and differential expression analysis. See Additional files 1, 2, 3 and 4 for cane toad counts and group labels.

### Data simulation

We simulated two sequencing experiments with two groups of 80 samples each (i.e., 160 samples total per experiment) using a forked version of polyester, hosted on GitHub, that adds multi-core support (archived at https://github.com/kcha/polyester/commits/545e33c9776db2927f9a22c8c2f5bfde2b3081a7).

To run polyester, we used the human GRCh37 DNA primary assembly FASTA file and the GRCh37.87 annotation GTF file, as compiled into a FA file using the gffread command line tool [15]. We set the parameters to achieve 20x coverage with a mean fragment length of 300 bases. Transcripts were selected randomly to have different magnitudes of differential expression with weighted probability: 4-fold up-regulation (3% of transcripts), 2-fold up (7%), 1.5-fold up (9%), 1.5-fold down-regulation (6%), 2-fold down (3%), and 4-fold down (2%). Each sample had a random multiplicative weight applied to their library size (with a mean between-group difference of 0.20 with a within-group standard deviation of 0.05).

Otherwise, the two simulated experiments differ only in the mean-variance relationship underlying the negative binomial model. The first is a low variance data set built using the default size argument. The second is a high variance data set built using size = 1 such that the variance of the negative binomial model equals the mean plus the mean squared. We selected these size parameters based on the precedent set by the polyester authors in their flagship publication [36]. Altogether, the resultant libraries ranged from 44 million reads to 79 million reads (per individual read pair file).

Note that, following alignment and quantification (described below), we analyzed simulated data by randomly sub-sampling from the total populations to establish unique data sets with 2, 3, 5, 10, and 20 replicates per group. We repeated this procedure 20 times for each sample size. To keep computations tractable, we also randomly sampled the feature space to include only 10,000 transcripts per instance.

### Alignment and quantification

To maintain benchmark comparisons with Williams et al. [16], we used alignment and quantification protocols (for each of our four data sets) congruent with theirs. For the two simulated data sets and the empirical data set from Williams et al. [16], we performed alignment to the GRCh37 release of the Human genome. For the cane toad data set [37], we performed alignment to the multi-tissue reference transcriptome published by Richardson et al. [38]. Alignments were conducted using both STAR v2.5.2a [6] and Salmon v0.8 [14]. For STAR, we used the “Basic” two-pass mode to output BAM alignments to the Human genome and transcriptome. For Salmon, alignments were made to the transcriptome (built using gffread, as for the simulated data) across 100 bootstraps. For Salmon, we trialled both the SMEM-based lightweight-alignment approach (hereafter called slFMD) and the “quasi-mapping” approach (hereafter called slQUASI).

We quantified expression at the transcript-level for all data, and at the gene-level for the Williams et al. [16] data. For transcript-level expression, counts were estimated using salmon quant for the slFMD and slQUASI alignments, as well as for the STAR transcriptome alignment (hereafter called stsl). For gene-level expression, counts were estimated using the “GeneCounts” quant mode in STAR (i.e., using the STAR alignment; hereafter called stst). We then condensed transcript-level expression to gene-level expression using the tximport package (available for the R programming language) [39] with the argument type = “salmon” and a key built from the EnsDb.Hsapiens.v86 database (from Bioconductor) [40].

### Log-ratio transformations

The ALDEx2 package produces different results depending on the log-ratio transformation used. Two transformations available in ALDEx2 are the centered log-ratio (clr) [28] and inter-quartile log-ratio (iqlr) transformations [30]. For *D* genes (or transcripts), the clr-transformation is defined as the logarithm of the transcript counts for the i-th sample, **x**, divided by the geometric mean of all counts for that sample, *g*(**x**):

1 $$\begin{array}{@{}rcl@{}} \text{clr}(\mathbf{x}) = \left[ \ln\frac{x_{1}}{g(\mathbf{x})};...; \ln\frac{x_{D}}{g(\mathbf{x})} \right] \end{array} $$

The iqlr-transformation replaces the *g*(**x**) denominator term with the geometric mean of those transcripts within the inter-quartile range of variability (i.e., prior to transformation), *g*(*x*_*iqr*_):

2 $$\begin{array}{@{}rcl@{}} \text{iqlr}(\mathbf{x}) = \left[ \ln\frac{x_{1}}{g(\mathbf{x_{iqr}})};...; \ln\frac{x_{D}}{g(\mathbf{x_{iqr}})} \right] \end{array} $$

In the analysis of the simulated data, we also use what we call the multi-additive log-ratio (malr) transformation. This uses the identity of all equally expressed transcripts as a reference set. Although this transformation is only feasible here because we already know a priori which transcripts are differentially expressed, it provides a “best case scenario” for normalization against which to compare other transformations. This transformation replaces the *g*(**x**) denominator term with the geometric mean of equally expressed transcripts, *g*(*x*_*eq*_):

3 $$\begin{array}{@{}rcl@{}} \text{malr}(\mathbf{x}) = \left[ \ln\frac{x_{1}}{g(\mathbf{x_{eq}})};...; \ln\frac{x_{D}}{g(\mathbf{x_{eq}})} \right] \end{array} $$

In addition, we introduce a novel transformation called the iterative iqlr (iilr) transformation. The iilr-transformation begins with the familiar iqlr-transformation, but then uses the results of a complete ALDEx2 analysis to inform a subsequent iteration of ALDEx2. After the initial iqlr-transformation, each new ALDEx2 run uses the geometric mean of the equally expressed transcripts identified by the prior ALDEx2 run. In principle, the equally expressed transcripts identified by each new iteration of ALDEx2 should more closely approximate the idealized *x*_*eq*_ used by the malr. We trial a single iteration of the iilr-transformation (ii1) as well as an approach using five iterations (ii5). In preparing this manuscript, we also contributed code to the ALDEx2 package to make the iilr transformation available by providing the argument test = “iterative” to the aldex function.

### Differential expression analysis

For each data set, and for each alignment and quantification protocol, we performed differential expression using the edgeR [18], DESeq2 [41], and ALDEx2 [30] packages (available for the R programming language). For the simulated data, we evaluated the performance of all differential expression methods using transcript-level abundances. For the Williams et al. [16] data, we also used gene-level abundances.

When applying ALDEx2 to the simulated data, we performed DE analysis with each combination of parameters: non-filter versus filter (i.e., the removal of transcripts without at least 10 counts in at least 20 samples), 8 versus 128 Monte Carlo instances, and clr versus iqlr versus malr versus iilr transformation. When applying ALDEx2 to the real data, we used the “non-filter” procedure with 128 Monte Carlo instances. For ALDEx2, we considered an expected Benjamini-Hochberg (FDR) adjusted *p*-value of the Wilcoxon Rank Sum test (i.e., column “wi.eBH”) less than 0.05 significant. We also repeated this procedure using Welch’s t-test (i.e., column “we.eBH”) (see Additional file 5: Figures). For clarity of visualization, all figures show results from “non-filter” runs with 128 Monte Carlo instances and the column “wi.eBH” (except where otherwise noted).

When applying edgeR to the simulated data, we performed DE analysis by applying these functions in order: calcNormFactors, estimateCommonDisp, estimateTagwiseDisp, and exactTest. When applying DESeq2 to the simulated data, we used the functions DESeqDataSetFromMatrix and DESeq. As above, we tested whether non-filter versus filter affects performance. For both methods, we considered an FDR-adjusted *p*-value less than 0.05 significant. For clarity of visualization, all figures show results from “non-filter” runs (except where otherwise noted).

When applying edgeR and DESeq2 to the Williams data, we used the protocols from Williams et al. [16]. When applying edgeR to the cane toad data, we used these same protocols. For both methods, we considered an FDR-adjusted *p*-value less than 0.05 significant.

### Performance estimates

For the simulated data, we calculated precision and recall from a contingency table of the simulated state of differential expression (as a binary) compared with the predicted state of differential expression (as a binary). For the Williams et al. [16] data, consistent with the original publication, we calculated precision and recall for each of the four microarray “truth sets” (available from the supplemental materials of [16]) separately, then reported the average precision and average recall [16].

Since the microarray “truth sets” were based on HGNC symbols, we needed to convert the aligned and quantified transcript-level and gene-level counts to HGNC-level counts; for this, we used code adapted from the Williams et al. methods in conjunction with a conversion table provided by the authors [16]. Using microarray “truth sets” also required an additional filter step to remove HGNC symbols detected by the microarray platform but not RNA-Seq (and *vice versa*). For this, we referenced the hgu133plus2.db, illuminaHumanv4.db, and illuminaHumanv2.db databases (from Bioconductor) to build an HGNC-level “gene universe” for each microarray platform [42]. We then performed a simple set intersection between the microarray “gene universe” with the RNA-Seq HGNC-level “gene universe” prior to calculating precision and recall. Note that our “gene universes” likely differ from those used by Williams et al. (which are not available from their Additional files) [16]. As such, any gene uniquely present (or absent) in our universe could marginally change the measured performance. Specifically, the numerator or denominator of the precision and recall estimates could change by an offset up to the number of genes uniquely present (or absent) in our universe.

We refer the reader to Additional file 6 for a table of all performances from the simulated data benchmark, and Additional file 7 for a table of all performances from the Williams et al. data benchmark. We make all scripts used in this analysis available in Additional file 8.

## Results

In order to evaluate the performance of ALDEx2 as a differential expression (DE) method for RNA-Seq data, we tested its performance on four data sets using several combinations of run-time parameters. Specifically, we assessed how changes in the alignment and quantification process, sample size, and log-ratio transformation affect the precision and recall of DE analysis. We also performed a DE analysis using edgeR and DESeq2 to provide a point of reference.

### ALDEx2 performance on a low variance simulated data set

Before generating any figures, we tested whether some of the run-time parameters (i.e., non-filter versus filter and 8 versus 128 Monte Carlo instances) impacted ALDEx2 performance (across all alignment and quantification procedures, log-ratio transformations, and sample sizes). We found that filtering the data before DE analysis did not change precision or recall for ALDEx2, nor did increasing the number of Monte Carlo instances to 128 (all unadjusted *p*>0.01 by *t*-test) [for low variance data, filtered or not]. Filtering the data before DE analysis also did not change precision or recall for edgeR and DESeq2 (all unadjusted *p*>0.01 by *t*-test) [for low variance data, regardless of the number of instances]. For clarity of visualization, all figures show results from “non-filter” runs with 128 Monte Carlo instances and the column “wi.eBH” (except where otherwise noted).

**Fig. 1:**
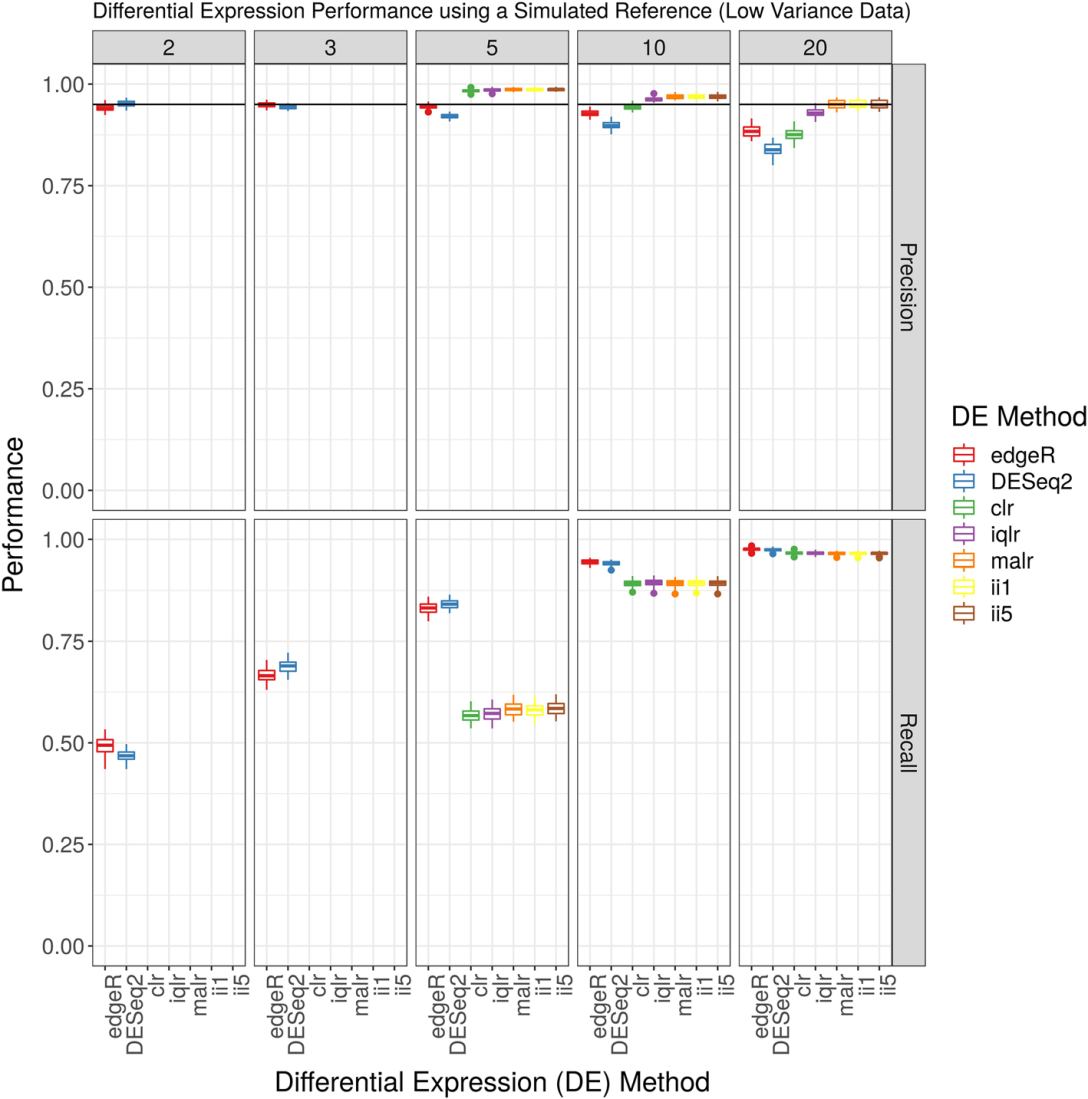
Differential expression analysis of low variance simulated data. This figure shows the performance (y-axis) of a complete differential expression analysis, organized by differential expression method (x-axis) and the number of replicates per group (panel). The acronyms clr, iqlr, malr, ii1, and ii5 describe log-ratio transformations (see Methods). The acronyms slFMD, slQUASI, and stsl describe alignment and quantification procedures (see Methods). Missing data suggest that the method did not call any transcripts differentially expressed (and therefore has no precision or recall). The horizontal line indicates a precision of 0.95, equivalent to the requested false discovery rate (FDR) of 0.05

**Fig. 2:**
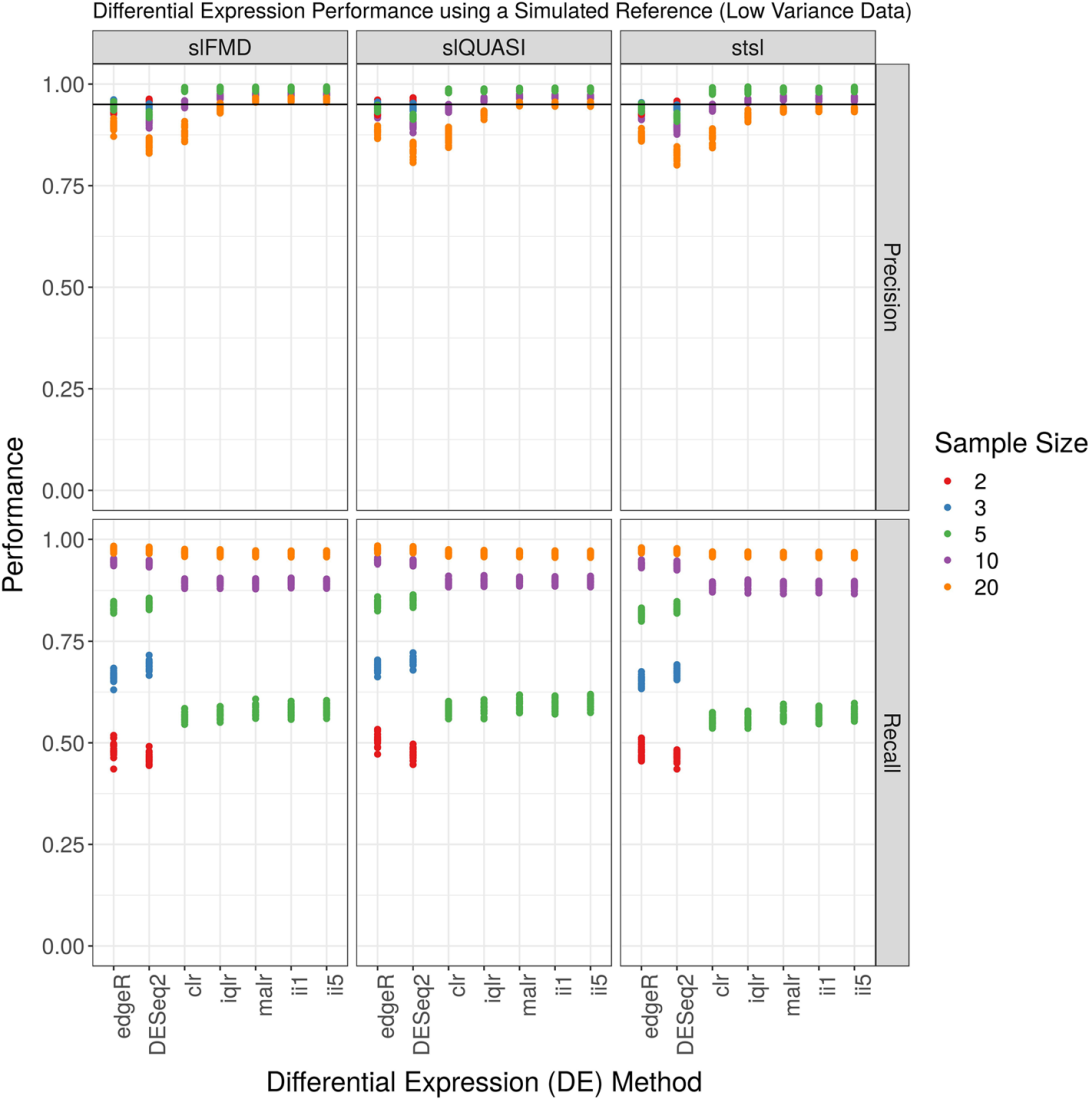
Differential expression analysis of low variance simulated data. This figure shows the performance (y-axis) of a complete differential expression analysis, organized by differential expression method (x-axis) and alignment and quantification procedure (panel). The acronyms clr, iqlr, malr, ii1, and ii5 describe log-ratio transformations (see Methods). The acronyms slFMD, slQUASI, and stsl describe alignment and quantification procedures (see Methods). Missing data suggest that the method did not call any transcripts differentially expressed (and therefore has no precision or recall). Precision (top-panel) and recall (bottom-panel) appear largely unaffected by choice in the alignment and quantification procedure. The horizontal line indicates a precision of 0.95, equivalent to the requested false discovery rate (FDR) of 0.05

Figure 1 shows the precision (top panel) and recall (bottom panel) for a DE analysis of the low variance simulated data set as plotted as a function of software method and log-ratio transformation, organized by the number of replicates per group. When there are 5 replicates per group, clr-based and iqlr-based ALDEx2 is more precise than edgeR and DESeq2 (all *p*<0.0001 by *t*-test). When there are 10 or 20 replicates per group, iqlr-based ALDEx2 is even more precise than these three (all *p*<0.0001 by *t*-test). However, for 5 and 10 replicates per group, ALDEx2 has less recall than edgeR and DESeq2 (all *p*<0.0001 by *t*-test), but, for 20 replicates per group, has similar recall (though still significantly less; all *p*<0.0001 by *t*-test). When there are only 2 or 3 replicates per group, ALDEx2 does not make any DE calls, and therefore has no precision or recall (though a Wilcoxon Rank Sum test cannot find significant differences with so few replicates). Figure 2 shows another projection of these data, organized by the alignment and quantification procedure used. Here, it becomes clear that the choice between STAR and Salmon alignment has no apparent impact on the results of DE analysis. Note that Figs. 1 and 2 show results from a transcript-level, not gene-level, analysis. We refer the reader to the Additional file 5: Figures for a replication of these figures using the column “we.eBH” from ALDEx2 (which improves recall when there are 3 or 5 replicates per group), as well as empiric false discovery rates (FDR) for low variance data. For these low variance data, all methods control FDR below *α*=0.05, although ALDEx2 appears to control FDR better than edgeR and DESeq2.

### ALDEx2 performance on a high variance simulated data set

**Fig. 3:**
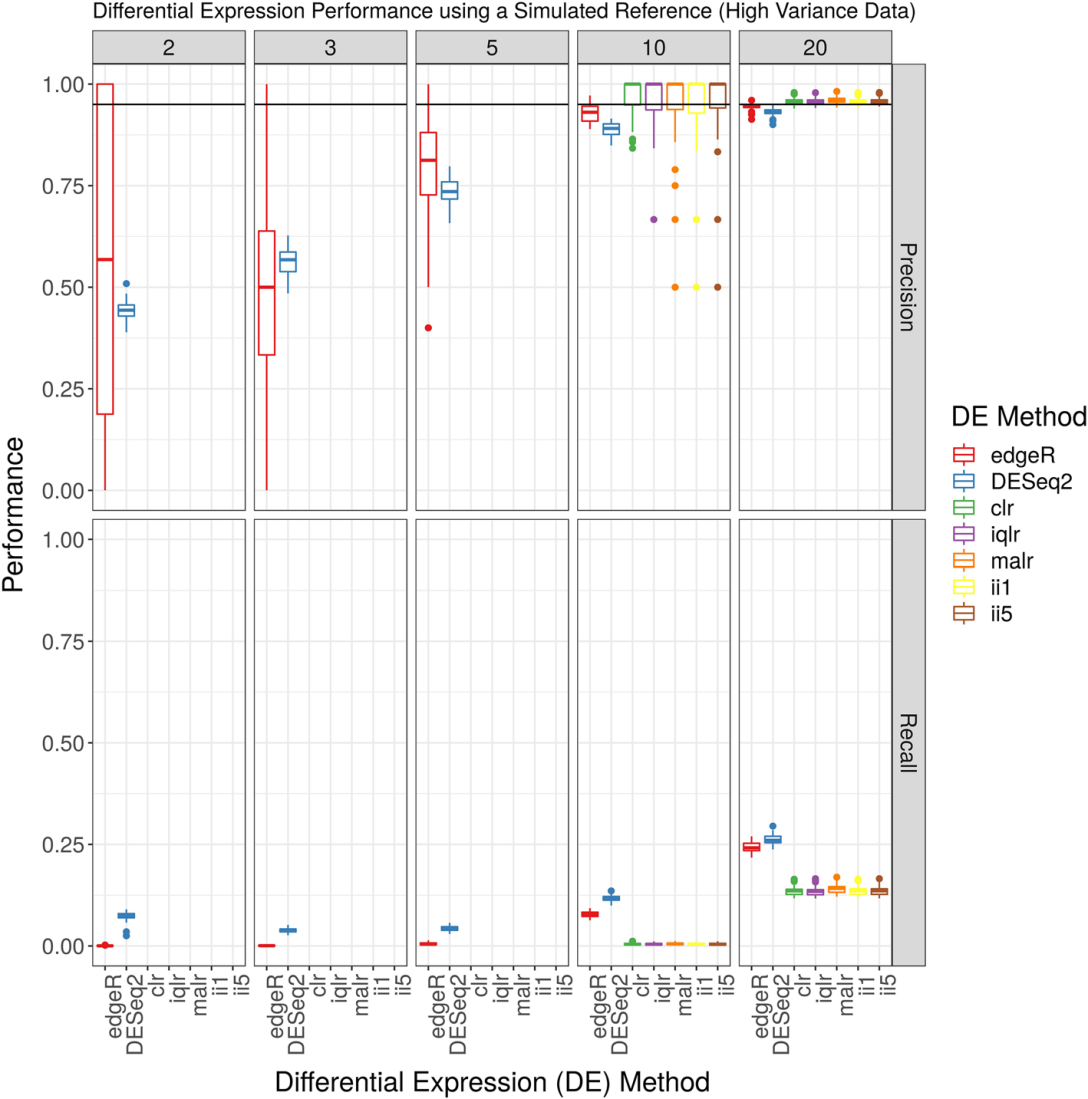
Differential expression analysis of high variance simulated data. This figure shows the performance (y-axis) of a complete differential expression analysis, organized by differential expression method (x-axis) and the number of replicates per group (panel). The acronyms clr, iqlr, malr, ii1, and ii5 describe log-ratio transformations (see Methods). The acronyms slFMD, slQUASI, and stsl describe alignment and quantification procedures (see Methods). Missing data suggest that the method did not call any transcripts differentially expressed (and therefore has no precision or recall). The horizontal line indicates a precision of 0.95, equivalent to the requested false discovery rate (FDR) of 0.05

Figure 3 reproduces Fig. 1 for a DE analysis of the high variance simulated data, organized by the number of replicates per group. Maximum recall rates less than 0.40 (even with 20 replicates per group) suggest that the data are indeed extremely variable. When there are 5 or less replicates per group, edgeR and DESeq2 have poor precision, while ALDEx2 does not make any DE calls (and therefore has no precision or recall). When there are 10 replicates per group, ALDEx2 tends to have higher median, but more variable, precision when compared with edgeR and DESeq2. Across all sample sizes, edgeR and DESeq2 outperform ALDEx2 in terms of recall. Note that Fig. 3 shows results from a transcript-level, not gene-level, analysis. We refer the reader to the Additional file 5: Figures for a replication of these figures using the column “we.eBH” from ALDEx2 (which does not seem to improve ALDEx2 performance for high variance data), as well as empiric false discovery rates (FDR) for high variance data. For these high variance data, edgeR and DESeq2 have an FDR above *α*=0.05.

### ALDEx2 performance on a previously benchmarked data set

**Fig. 4:**
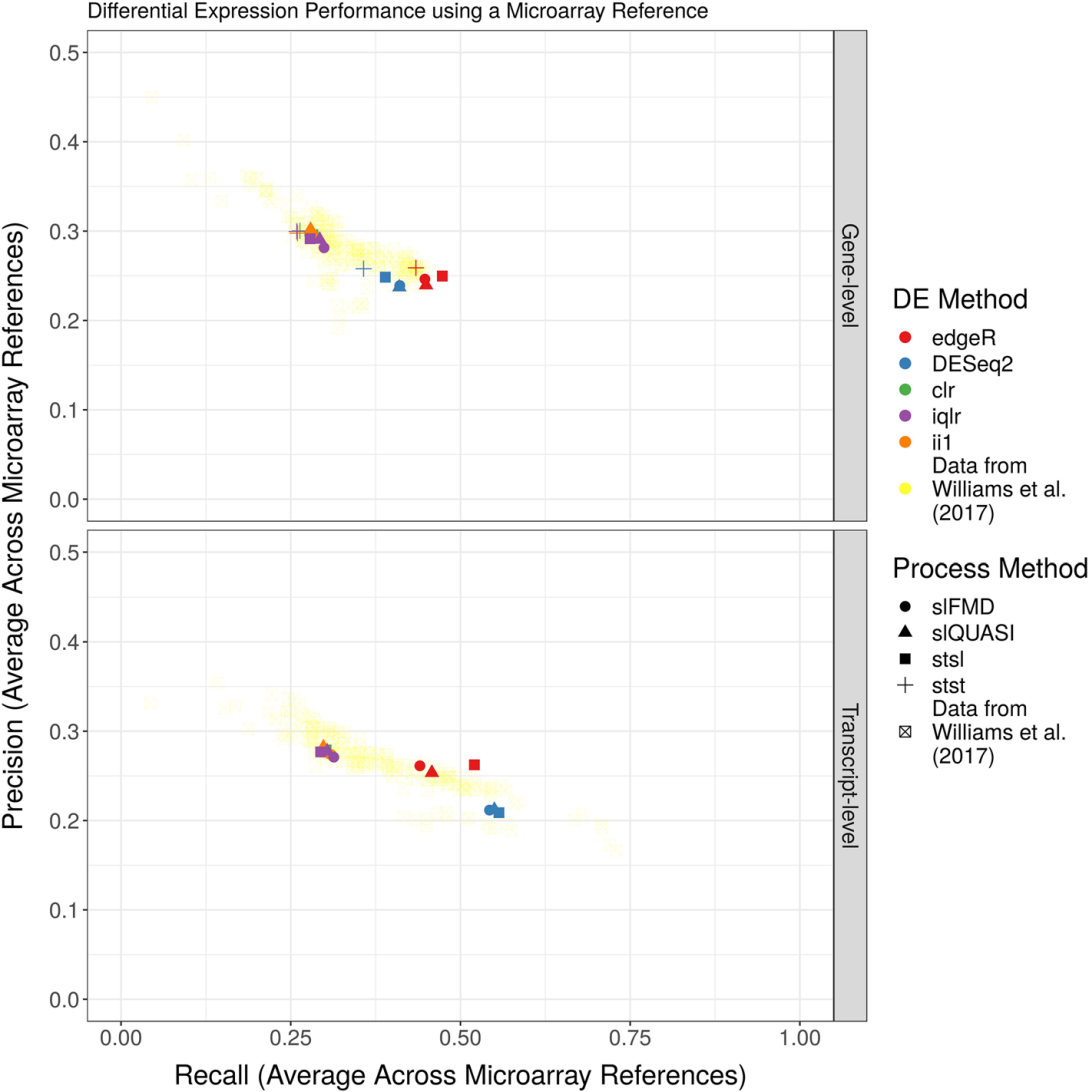
Differential expression analysis of Williams et al. data. This figure shows the precision (y-axis) versus the recall (x-axis) of a complete differential expression analysis applied to real RNA-Seq data. The “truth set” is established using a microarray reference (see Methods). The acronyms clr, iqlr, and ii1 describe log-ratio transformations (see Methods). The acronyms slFMD, slQUASI, stsl, and stst describe alignment and quantification procedures (see Methods). Translucent data points show performance calculated from a previously published systematic benchmark. ALDEx2 tends toward higher precision and lower recall

Figure 4 shows precision (y-axis) versus recall (x-axis) for a gene-level (top panel) and transcript-level (bottom panel) DE analysis of the Williams et al. RNA-Seq data (that uses pooled microarray data as a “truth set”) [16]. Here, the smear of lightly colored transparent points indicate the precision and recall recorded by the original Williams et al. publication (sourced from their Additional files) [16]. Meanwhile, the dark opaque dots indicate the precision and recall measured during our replication of their procedure. Compared with a myriad of other alignment, quantification, and DE method combinations (including edgeR and DESeq2), ALDEx2 tends toward higher precision and lower recall, especially for the gene-level analysis. Interestingly, differences between the choice of log-ratio transformation appear unimportant for these data. Note that the multiplicity of points for each DE method represent different alignment and quantification procedures.

### Agreement between ALDEx2 and edgeR

**Fig. 5:**
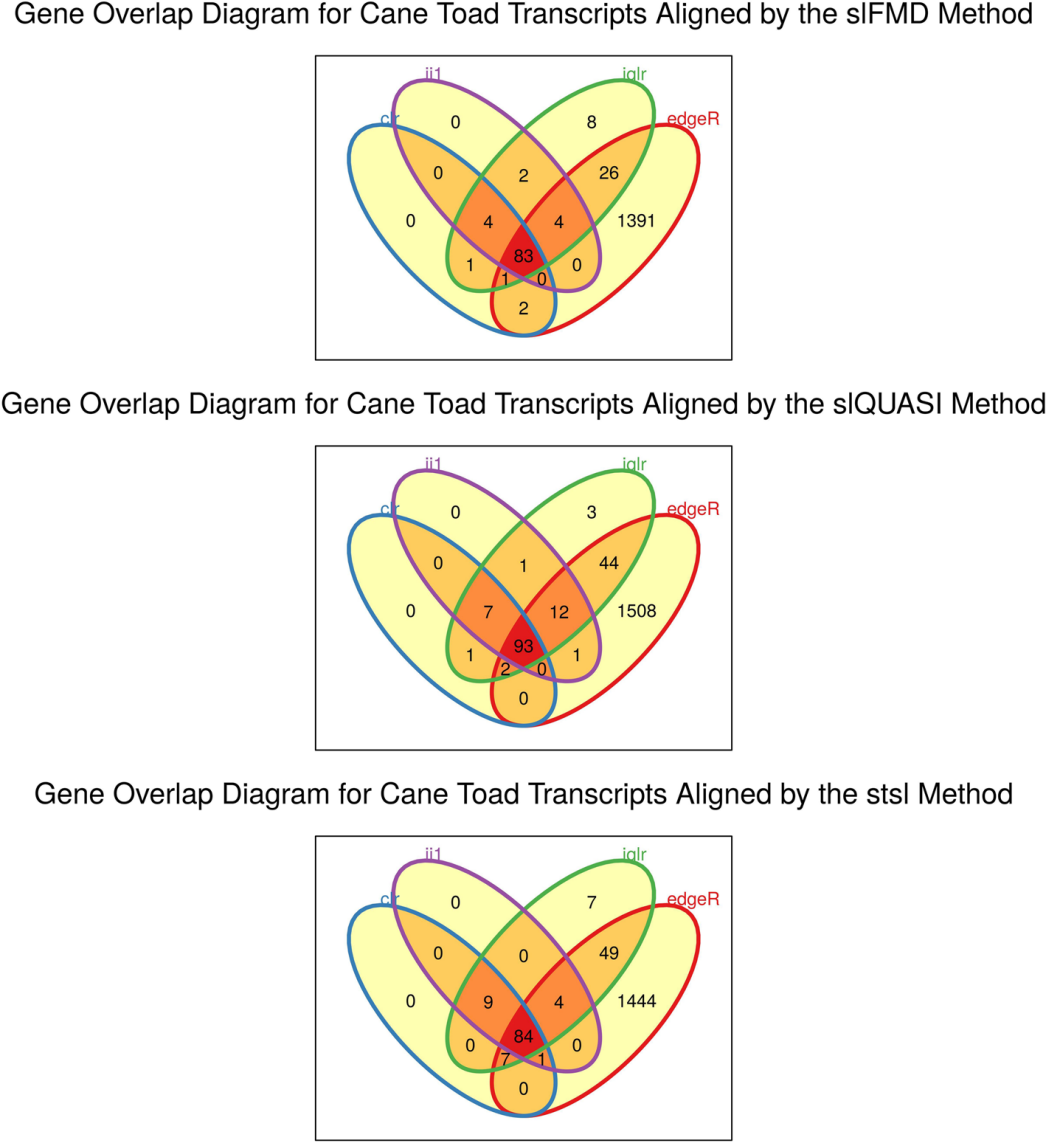
Gene overlap diagrams of Rollins et al. data. This figure shows the intersection of differentially expressed transcripts selected by four differential expression methods (organized by the alignment and quantification procedure used). The acronyms clr, iqlr, and ii1 describe log-ratio transformations (see Methods). The acronyms slFMD, slQUASI, and stsl describe alignment and quantification procedures (see Methods). Differentially expressed transcripts selected based on an FDR < 0.05 as calculated by the respective method. Figure prepared from Rollins et al. data

Figure 5 shows the intersection of differentially expressed transcripts selected by four differential expression methods (organized by the alignment and quantification procedure used) using the Rollins et al. [37] RNA-Seq data. These data derive from a study of the transcriptomic differences between two wild populations of cane toads (with 10 samples in each group). Here, we compare ALDEx2 with edgeR (i.e., the method used in the original publication), and find that the overwhelming number of transcripts called differentially expressed by ALDEx2 (regardless of the log-ratio transformation used) are also called differentially expressed by edgeR. However, edgeR calls many transcripts differentially expressed that ALDEx2 does not. This is consistent with the prior benchmarking that suggests while both methods have high precision, edgeR tends to have higher sensitivity, especially for data with 10 or less replicates per group.

**Fig. 6:**
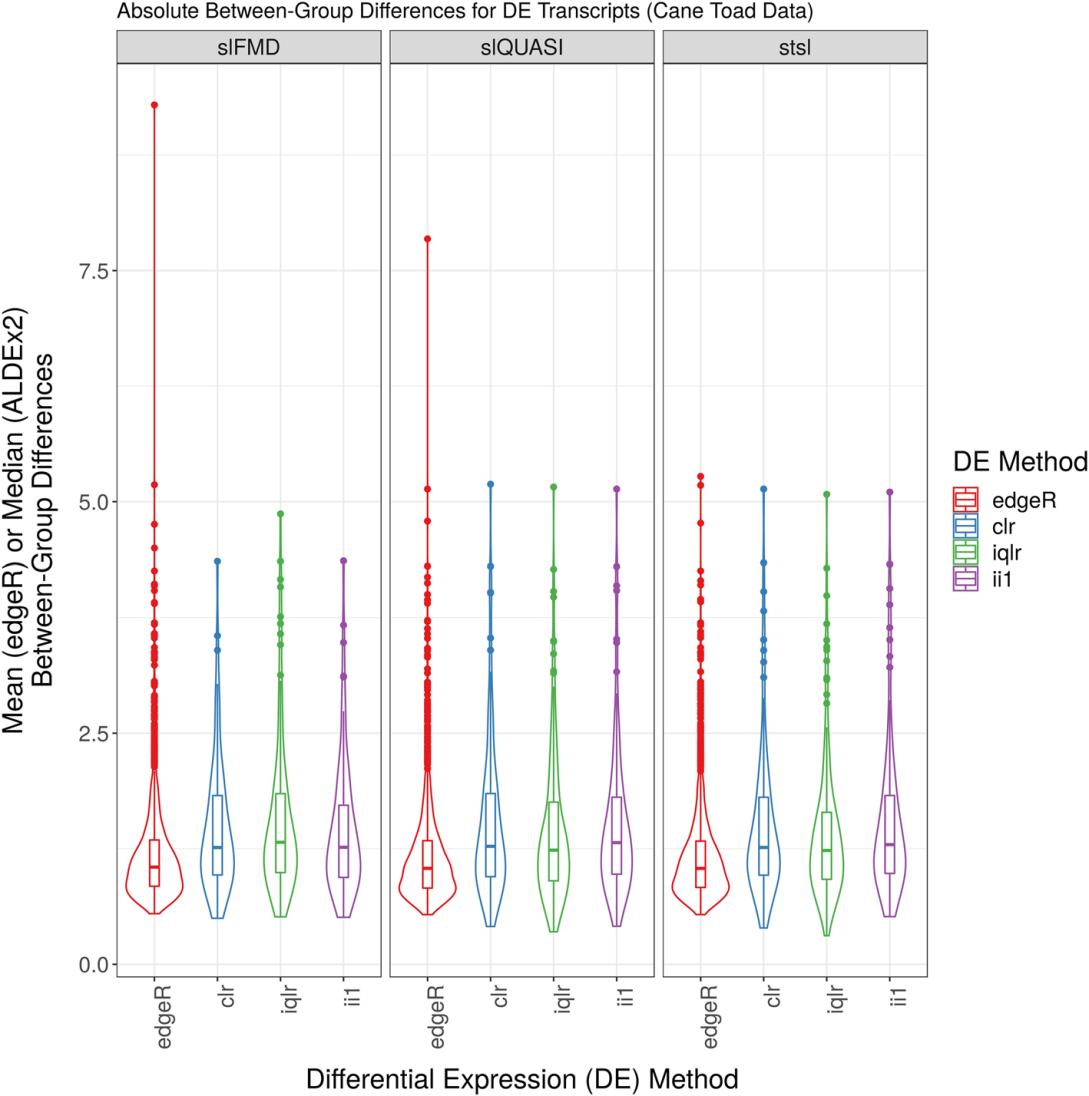
Between-group differences of Rollins et al. data. This figure shows the mean (or median) absolute between-group differences for differentially expressed transcripts (y-axis) versus the differential expression method (x-axis) (organized by the alignment and quantification procedure used). Between-group differences are reported as measured by the respected method (i.e., mean for edgeR and median for ALDEx2). The acronyms clr, iqlr, and ii1 describe log-ratio transformations (see Methods). The acronyms slFMD, slQUASI, and stsl describe alignment and quantification procedures (see Methods). Differentially expressed transcripts selected based on an FDR < 0.05 as calculated by the respective method. Figure prepared from Rollins et al. data

**Fig. 7:**
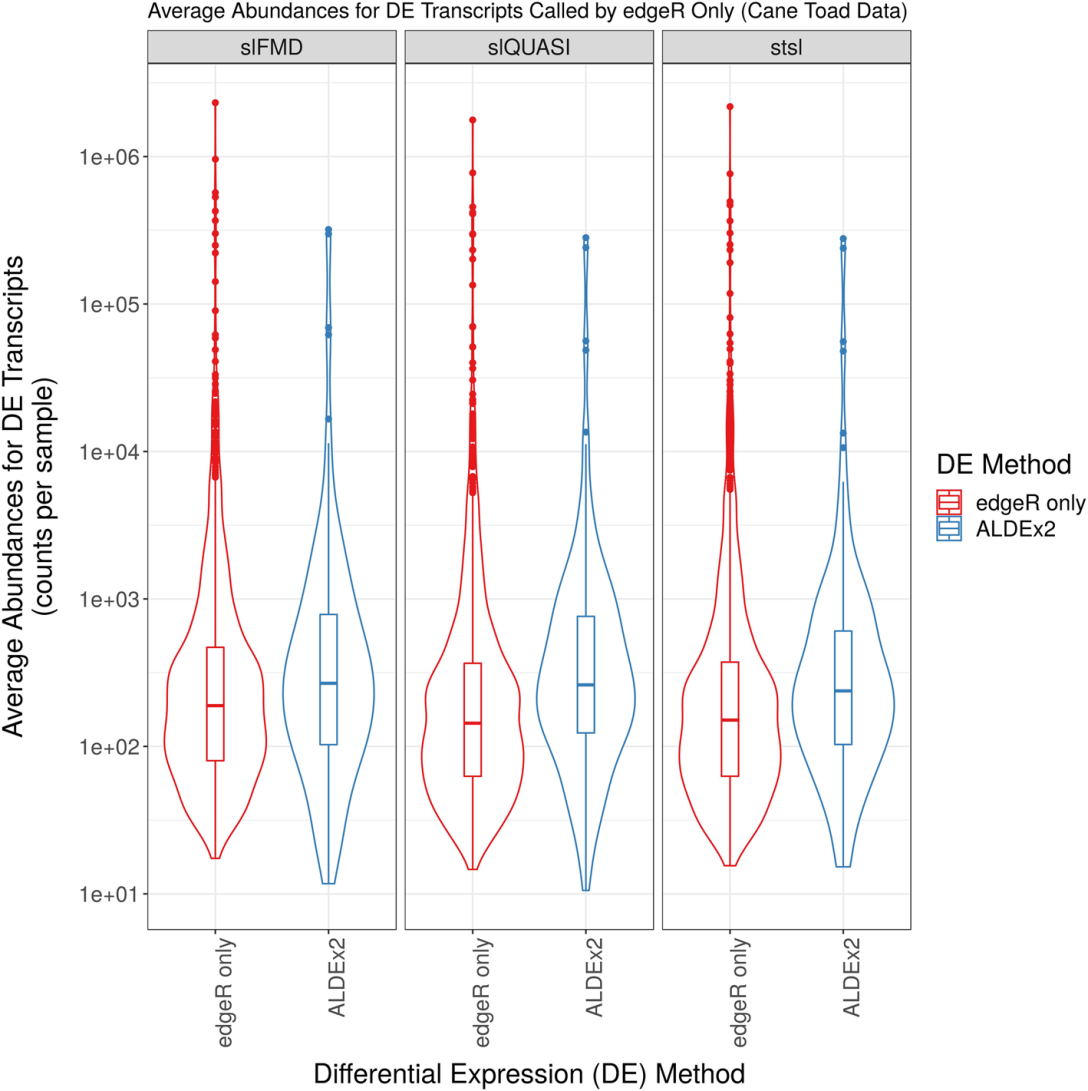
Mean relative abundance of transcripts missed by ALDEx2. This figure shows the mean relative abundance for differentially expressed transcripts (y-axis) versus the differential expression method (x-axis) (organized by the alignment and quantification procedure used). Mean relative abundances are provided for all transcripts selected by either (a) any ALDEx2 method (regardless of log-ratio transformation used) or (b) edgeR method only (and not any ALDEx2 method). The acronyms slFMD, slQUASI, and stsl describe alignment and quantification procedures (see Methods). Differentially expressed transcripts selected based on an FDR < 0.05 as calculated by the respective method. Figure prepared from Rollins et al. data

Figure 6 shows the mean (or median) absolute between-group differences for differentially expressed transcripts (y-axis) versus the differential expression method (x-axis) (organized by the alignment and quantification procedure used). Between-group differences are reported as measured by the respective method (i.e., mean for edgeR and median for ALDEx2). This figure suggests that both methods can detect between-group differences at approximately the same threshold. We interpret this to mean that the decreased sensitivity of ALDEx2 is not easily explained by an inability to detect small-fold differences in expression between groups (although edgeR does appear to detect *more* small-fold differences). Figure 7 shows the average number of counts for each of the transcripts called DE by any ALDEx2 compared with those called DE by *only*edgeR (organized by the alignment and quantification procedure used). This suggests that ALDEx2 tends to “miss” DE among transcripts with the least relative abundance.

## Discussion

### ALDEx2 has high precision and variable recall for RNA-Seq data

The ALDEx2 package, most often used to detect differential abundance in 16S rRNA data, has received extensive use for that purpose (e.g., [33, 34]). In previous studies, ALDEx2 was shown to produce low false discovery rates (FDR) for highly sparse compositional data [35] (FDR=1−precision). However, we have not yet encountered a study that independently evaluates ALDEx2 as a differential expression (DE) analysis method for RNA-Seq data (excepting a manuscript by the ALDEx2 authors which defended its use for RNA-Seq data [32]).

In general, our analysis of simulated and real data agrees with Fernandes et al. [32]: ALDEx2 can accurately identify differentially expressed genes (and transcripts) in RNA-Seq data. Specifically, ALDEx2 identifies differentially expressed genes with high precision (i.e., few false positives), but can suffer from low recall (i.e., many false negatives) in the setting of small sample sizes. Overall, based on our simulations, we find that ALDEx2 performs best when there are at least 10, but ideally 20, replicates per group.

We offer three explanations for the low recall. First, ALDEx2 uses non-parametric statistical modeling which tend to have reduced power for small RNA-Seq studies [16, 43] (though, the package authors note that log-ratio transformed data do not necessarily adhere to a normal distribution [32]). Indeed, using the column “we.eBH” (a parametric alternative to the column “wi.eBH”) improves recall when there are only 3 or 5 replicates per group (see Additional file 5: Figures). Second, methods like edgeR use an empiric Bayes method that “shares information between genes” to shrink per-gene variance estimates and improve power [18]. Presumably, ALDEx2 would perform better if one could extend moderation to its transformation-based analysis. Third, ALDEx2 generates models of the data by drawing from the Dirichlet distribution. As such, it is not deterministic, and transcripts close to the margin of significance may not get called DE. This is supported by Fig. 7 which suggests that transcripts called DE by edgeR only (and not ALDEx2) have, on average, lower counts than those called DE by ALDEx2.

Throughout this benchmarking exercise, we had the opportunity to see how two run-time parameters affect ALDEx2 performance. First, we noted that the removal of lowly abundant counts does not impact performance (all unadjusted *p*>0.01 by *t*-test) (see Additional file 6: Tables), nor did it affect edgeR or DESeq2. Second, we noted that ALDEx2 performs almost as well using only 8 Monte Carlo instances when compared with using 128 Monte Carlo instances (all unadjusted *p*>0.01 by *t*-test) (see Additional file 6: Tables). Although the package vignette recommends “128 or more mc.samples for the t-test”, this change improves run-time 16-fold.

### ALDEx2 performance does not depend on alignment and quantification used

Across all four data sets used in this study, the choice in the alignment and quantification procedure did not change the overall performance of differential expression analysis by ALDEx2 (or edgeR or DESeq2). This even holds true for the Salmon “quasi-mapping” method that runs many-fold times faster than other quantification algorithms [44]. Although the computational basis of “quasi-mapping” differs from other approaches, this method produces (pseudo-)counts that appear to work well for trimmed M of means (TMM) normalization (used by edgeR) and log-ratio transformation (used by ALDEx2) alike. Broadly speaking, our results agree with the Williams et al. paper in that the choice in differential expression method matters more than the choice in the alignment and quantification method [16].

### ALDEx2 performance depends on log-ratio transformation used

First of all, it is necessary to emphasize that, although log-ratio transformations can be used in lieu of normalization, such transformations do not formally reclaim absolute abundances from relative abundances (see [29]). Yet, benchmarking a transformation-based analysis against a “truth set” implies that the transformation is interpreted as if it were a normalization (i.e., that the reference denominator used for the transformation has rescaled the data to absolute terms [29]). In other words, the more that the reference approximates a feature with fixed abundance across all samples, the more that the transformed data resemble the absolute data. Therefore, the benchmarked performance of a log-ratio transformation-based analysis depends on whether the reference denominator of the transformation is an ideal reference with fixed abundance.

When interpreting the clr transformation as if it were a normalization, there is an implicit assumption that the majority of genes (or transcripts) are not differentially expressed [21]. Meanwhile, the iqlr transformation would assume that a portion of genes (i.e., those with their variances within the inter-quartile range) are not differentially expressed. Likewise, the iterative transformation assumes that the results of an ALDEx2 analysis selects (non-)differentially expressed genes more accurately than a simpler transformation. Therefore, all else being equal, one can interpret the performance of each transformation as a proxy for how well it reclaims absolute information (i.e., how well it approximates an ideal reference with fixed abundance).

For simulated data, some transformations perform better than others. First, the iqlr transformation is more precise than the clr transformation. This is expected because the iqlr transformation should be more robust to imbalances in up- or down- regulation [45]. Second, the iterative transformations is more precise than the iqlr, suggesting that novel log-ratio transformations could improve performance beyond those routinely used. Perhaps surprisingly, this trend was not apparent among the Williams et al. [16] data: there were no impressive differences in precision or recall between transformation-based methods. This could be due to either the absence of any imbalance in this data set (such that iqlr and others offer no clear benefit over clr) or limitations in the microarray-based “truth set” (which may not have controlled for a systematic imbalance in the biological samples). However, for the Rollins et al. [37] data, the iqlr-based analysis not only agreed most with edgeR, but also identified the greatest number of differentially expressed transcripts, suggesting an advantage to using the iqlr transformation (although the lack of a truth set makes it impossible to quantify precision and recall directly).

### Limitations of ALDEx2 and other transformation-based methods

Users may encounter some practical limitations with ALDEx2 as it is currently implemented. First, owing to the replication of non-parametric analyses across multiple Monte Carlo instances, ALDEx2 runs much slower than edgeR and other methods (though in preparing this manuscript we submitted code to speed up the software). Second, ALDEx2 does not contain a well-documented generalization to mixed models. Although this package does have a multi-group procedure (i.e., using Kruskal-Wallis), it takes even longer to run than the standard two-group comparisons.

There are also limitations that come with interpreting the log-ratio transformation as a kind of normalization. As mentioned above, using the clr in this way assumes that the majority of genes (or transcripts) are not differentially expressed [21], the same assumption held by the trimmed mean of M (TMM) normalization [20]. When this assumption does not hold, it is not possible to infer absolute differences in expression between samples. As such, both will fail if one of the experimental groups has massively more up-regulation than down-regulation (or *vice versa*). This scenario is exemplified by a cell line with high levels of c-Myc, a protein with widely variable expression in tumors that can amplify gene expression to produce 2-3 times more total RNA [46]. When comparing high c-Myc cells against low c-Myc cells, a standard RNA-Seq analysis would conclude that there is both up- and down-regulation, even though the cells actually only have up-regulation (as determined by using spike-in controls normalized to cell number) [46]. A transformation-based approach would not fair any better here, unless one was exclusively interested in identifying transcripts that were differentially expressed *relative to a reference* [29]. Newer methods that avoid normalization and transformation altogether by analyzing transcript ratios directly may serve as an alternative in these cases [47, 48].

Finally, ALDEx2 is said to suffer from another limitation based on how it uses the Dirichlet distribution to generate Monte Carlo instances. Weiss et al. note, “this formulation assumes a Dirichlet-multinomial framework, which imposes a negative correlation structure on every pair of [features]” [49]. In fact, Hawinkel et al. showed that, when simulating 16S relative abundance data to have a positive correlation structure, ALDEx2 results depart from the uniformity of the *p*-value distribution (in the liberal direction) and show an increase in the nominal false discovery rate (FDR) (although FDR-adjustment still brought FDR below the 0.05 threshold) [50]. This publication also found that a positive correlation structure negatively impacted the performance of popular differential expression analysis methods like DESeq2 and edgeR (the latter of which had very high FDR-adjusted FDR rates) [50]. Yet, criticizing the Dirichlet-based framework for this reason is problematic because, by definition, all compositional data have a negative correlation bias that is different than the correlation structure of the underlying absolute abundances [51]. Nevertheless, we find that, for technical replicates of simulated data, ALDEx2 better controls false discoveries at *α*=0.05 than either edgeR or DESeq2, and in all cases has an FDR less than *α* (see Additional file 5: Figures).

## Conclusions

Using simulated and real RNA-Seq data, we find that the ALDEx2 package has very high precision in identifying differentially expressed genes (and transcripts). With enough replicates per group, it also has good recall. Consistent with other benchmark studies, we also find that the choice in the alignment and quantification procedure does not seem to affect performance, freeing investigators to choose a method based on convenience. Yet, when interpreting log-ratio transformations as if they were normalizations, some transformations work better than others: the inter-quartile range log-ratio (iqlr) transformation tends to out-perform the centered log-ratio (clr) transformation, likely owing to its ability to correct for some data asymmetry. We recommend using the iqlr transformation as the default setting when using ALDEx2, unless researchers have already established a known reference set that makes normalization with a (multi-)additive log-ratio (malr) transformation preferable. Altogether, our results suggest that ALDEx2, as a log-ratio transformation-based method, given a sufficient number of replicates, is appropriate for the analysis of RNA-Seq data. With previously demonstrated applicability to 16S rRNA data, ALDEx2 can thus serve as a single tool for data from multiple sequencing modalities.

## Additional files

Additional file 1 Estimated relative abundance for Rollins et al. data (by slFMD method). This table contains the relative transcript abundances as estimated using the slFMD procedure. (CSV 6166 kb)

Additional file 2 Estimated relative abundance for Rollins et al. data (by slQUASI method). This table contains the relative transcript abundances as estimated using the slQUASI procedure. (CSV 4758 kb)

Additional file 3 Estimated relative abundance for Rollins et al. data (by stsl method). This table contains the relative transcript abundances as estimated using the stsl procedure. (CSV 4579 kb)

Additional file 4 Group labels for Rollins et al. data. This table contains the group labels for all samples from the Rollins et al. cane toad data set. (CSV 1 kb)

Additional file 5 Supplemental figures. This file contains all supplemental figures referenced in this document. (PDF 7488 kb)

Additional file 6 Simulated data benchmark performance. This table contains the precision and recall estimates for several methods as applied to the low and high variance simulated data. This table is used to make figures. (CSV 2945 kb)

Additional file 7 Benchmark performance for Williams et al. data. This table contains the precision and recall estimates for several methods as applied to the Williams et al. data. This table is used to make figures. (CSV 6 kb)

Additional file 8 Analysis scripts. This file contains all the scripts used to generate the simulated data, benchmark methods on the simulated data, benchmark methods on the real data, parse the results, and make the figures. (PDF 3948 kb)

## Abbreviations

clr: centered log-ratio
CoDA: Compositional data analysis
DA: Differential abundance
DE: Differential expression
FDR: False discovery rate
ii1: One iteration of iterative inter-quartile log-ratio
ii5: Five iterations of iterative inter-quartile log-ratio
iilr: Iterative inter-quartile log-ratio
iqlr: Inter-quartile log-ratio
malr: Multi-additive log-ratio
MC: Monte Carlo
NGS: Next generation sequencing
OTUs: Operational taxonomic units
RNA-Seq: RNA-sequencing
slFMD: Salmon with SMEM-based lightweight-alignment
slQUASI: Salmon with “quasi-mapping”
stsl: STAR alignment with Salmon quantification
stst: STAR alignment with STAR quantification
TMM: Trimmed mean of M
