# Supplementary material for: Benchmarking differential expression analysis tools for RNA-Seq: normalization-based vs. log-ratio transformation-based methods
Source: BMC Bioinformatics. 2018 Jul 18;19:274. doi: 10.1186/s12859-018-2261-8 (PMC6052553; doi:10.1186/s12859-018-2261-8)
Supplement: Supplementary file 5 — Supplemental figures. This file contains all supplemental figures referenced in this document. (PDF 7488 kb) [file 12859_2018_2261_MOESM5_ESM.pdf]

Supplementary Information for Benchmarking differential expression analysis tools for RNA-Seq: normalization-based vs. log-ratio transformation-based methods

Thomas P. Quinn[1,2,*], Tamsyn M. Crowley[1,2,3], and Mark F. Richardson[2,4]

[1]Centre for Molecular and Medical Research, Deakin University, Geelong, 3220, Australia
[2]Bioinformatics Core Research Group, Deakin University, Geelong, 3220, Australia
[3]Poultry Hub Australia, University of New England, Armidale, New South Wales, 2351, Australia
[4]Centre for Integrative Ecology, Deakin University, Geelong, 3220, Australia
* *contacttomquinn@gmail.com*

Abstract

Background Count data generated by next-generation sequencing assays do not measure absolute transcript abundances. Instead, the data are constrained to an arbitrary "library size" by the sequencing depth of the assay, and typically must be normalized prior to statistical analysis. The constrained nature of these data means one could alternatively use a log-ratio transformation in lieu of normalization, as often done when testing for differential abundance (DA) of operational taxonomic units (OTUs) in 16S rRNA data. Therefore, we benchmark how well the ALDEx2 package, a transformation-based DA tool, detects differential expression in high-throughput RNA-sequencing data (RNA-Seq), compared to conventional RNA-Seq methods such as edgeR and DESeq2.

Results To evaluate the performance of log-ratio transformation-based tools, we apply the ALDEx2 package to two simulated, and two real, RNA-Seq data sets. One of the latter was previously used to benchmark dozens of conventional RNA-Seq differential expression methods, enabling us to directly compare transformation-based approaches. We show that ALDEx2, widely used in meta-genomics research, identifies differentially expressed genes (and transcripts) from RNA-Seq data with high precision and, given sufficient sample sizes, high recall too (regardless of the alignment and quantification procedure used). Although we show that the choice in log-ratio transformation can affect performance, ALDEx2 has high precision (i.e., few false positives) across all transformations. Finally, we present a novel, iterative log-ratio transformation (now implemented in ALDEx2) that further improves performance in simulations.

Conclusions Our results suggest that log-ratio transformation-based methods can work to measure differential expression from RNA-Seq data, provided that certain assumptions are met. Moreover, these methods have very high precision (i.e., few false positives) in simulations and perform well on real data too. With previously demonstrated applicability to 16S rRNA data, ALDEx2 can thus serve as a single tool for data from multiple sequencing modalities.

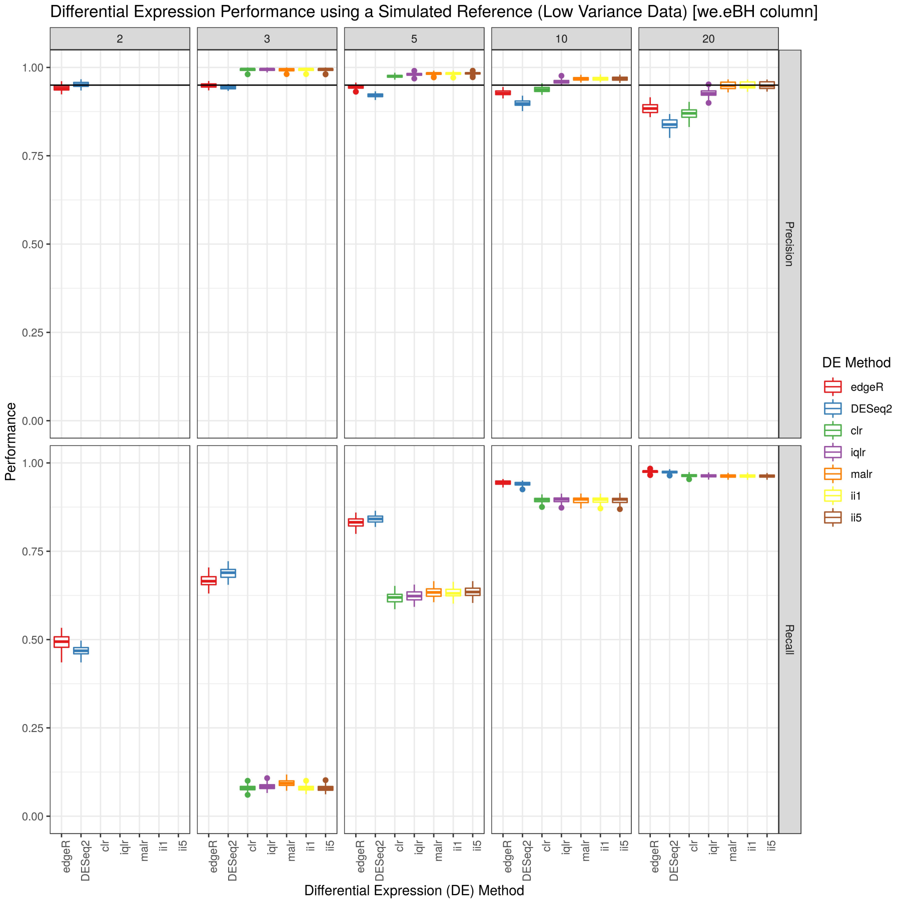

Figure 1: **Differential expression analysis of low variance simulated data.** This figure shows the performance (y-axis) of a complete differential expression analysis, organized by differential expression method (x-axis) and the number of replicates per group (panel). The acronyms clr, iqlr, malr, ii1, and ii5 describe log-ratio transformations (see Methods). The acronyms slFMD, slQUASI, and stsl describe alignment and quantification procedures (see Methods). Missing data suggest that the method did not call any transcripts differentially expressed (and therefore has no precision or recall). The horizontal line indicates a precision of 0.95, equivalent to the requested false discovery rate (FDR) of 0.05. This figure describes ALDEx2 performance based on the column "we.eBH".

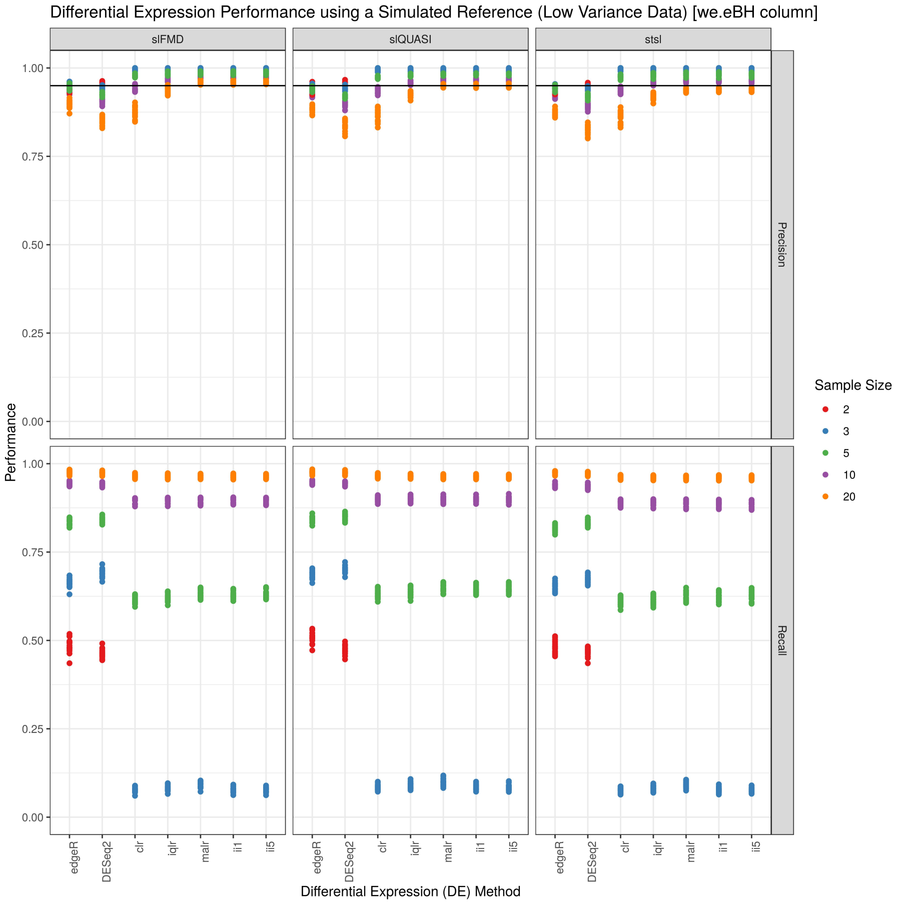

Figure 2: **Differential expression analysis of low variance simulated data.** This figure shows the performance (y-axis) of a complete differential expression analysis, organized by differential expression method (x-axis) and alignment and quantification procedure (panel). The acronyms clr, iqlr, malr, ii1, and ii5 describe log-ratio transformations (see Methods). The acronyms slFMD, slQUASI, and stsl describe alignment and quantification procedures (see Methods). Missing data suggest that the method did not call any transcripts differentially expressed (and therefore has no precision or recall). Precision (top-panel) and recall (bottom-panel) appear largely unaffected by choice in the alignment and quantification procedure. The horizontal line indicates a precision of 0.95, equivalent to the requested false discovery rate (FDR) of 0.05. This figure describes ALDEx2 performance based on the column "we.eBH".

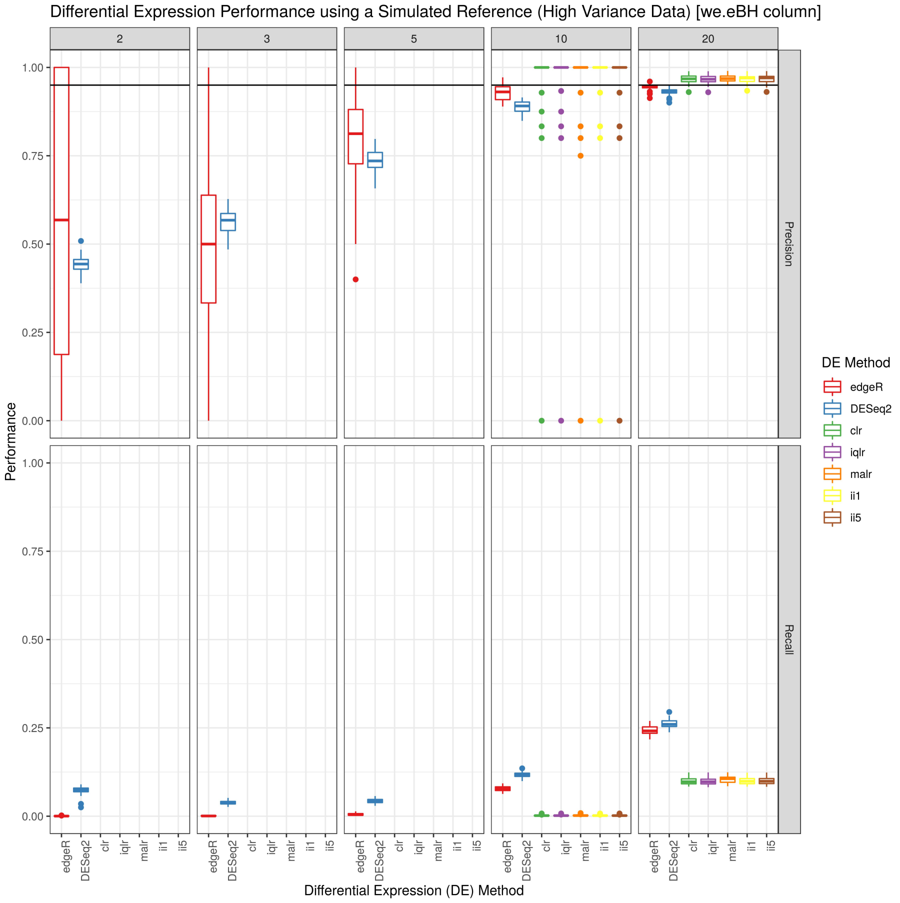

Figure 3: **Differential expression analysis of high variance simulated data.** This figure shows the performance (y-axis) of a complete differential expression analysis, organized by differential expression method (x-axis) and the number of replicates per group (panel). The acronyms clr, iqlr, malr, ii1, and ii5 describe log-ratio transformations (see Methods). The acronyms slFMD, slQUASI, and stsl describe alignment and quantification procedures (see Methods). Missing data suggest that the method did not call any transcripts differentially expressed (and therefore has no precision or recall). The horizontal line indicates a precision of 0.95, equivalent to the requested false discovery rate (FDR) of 0.05. This figure describes ALDEx2 performance based on the column "we.eBH".

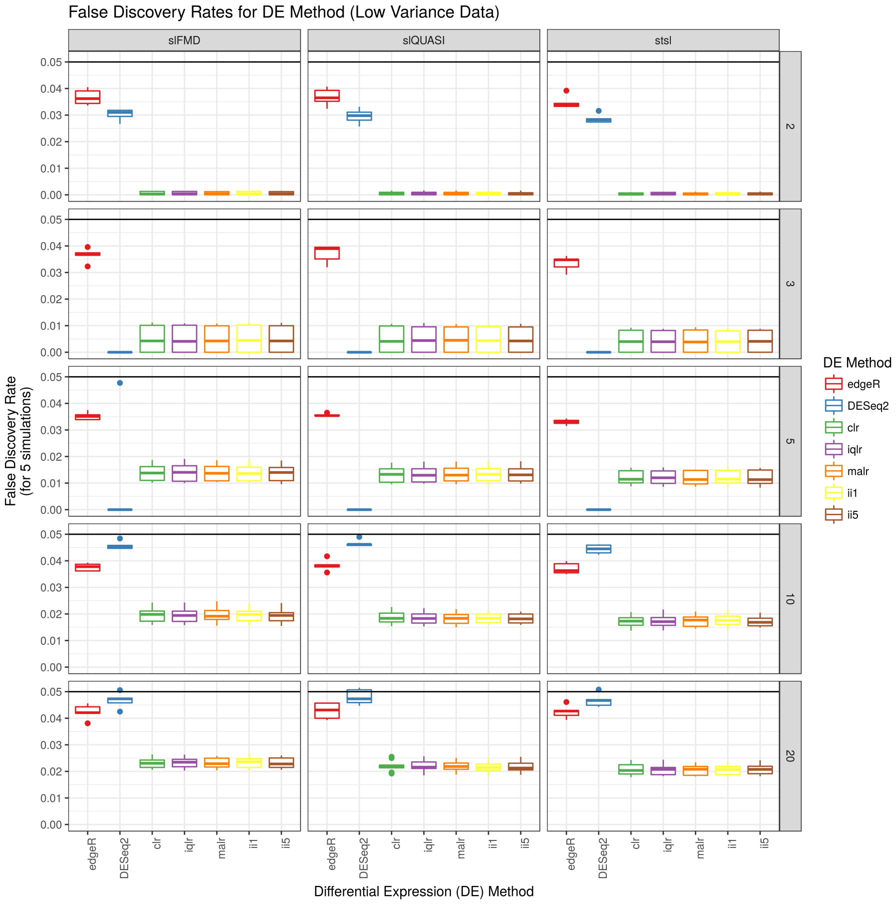

Figure 4: **FDR for the differential expression analysis of low variance data.** This figure shows the false discovery rate (FDR) (y-axis) versus the differential expression method (x-axis). FDR is calculated by analyzing two groups of replicates sampled from the same parent population (repeated 5 times). The results are further organized by alignment and quantification method, and the number of replicates per group. The acronyms clr, iqlr, malr, ii1, and ii5 describe log-ratio transformations (see Methods). The acronyms slFMD, slQUASI, and stsl describe alignment and quantification procedures (see Methods). For these low variance data, all methods control FDR below $\alpha = 0.05$, although ALDEx2 appears to control FDR better than edgeR and DESeq2.

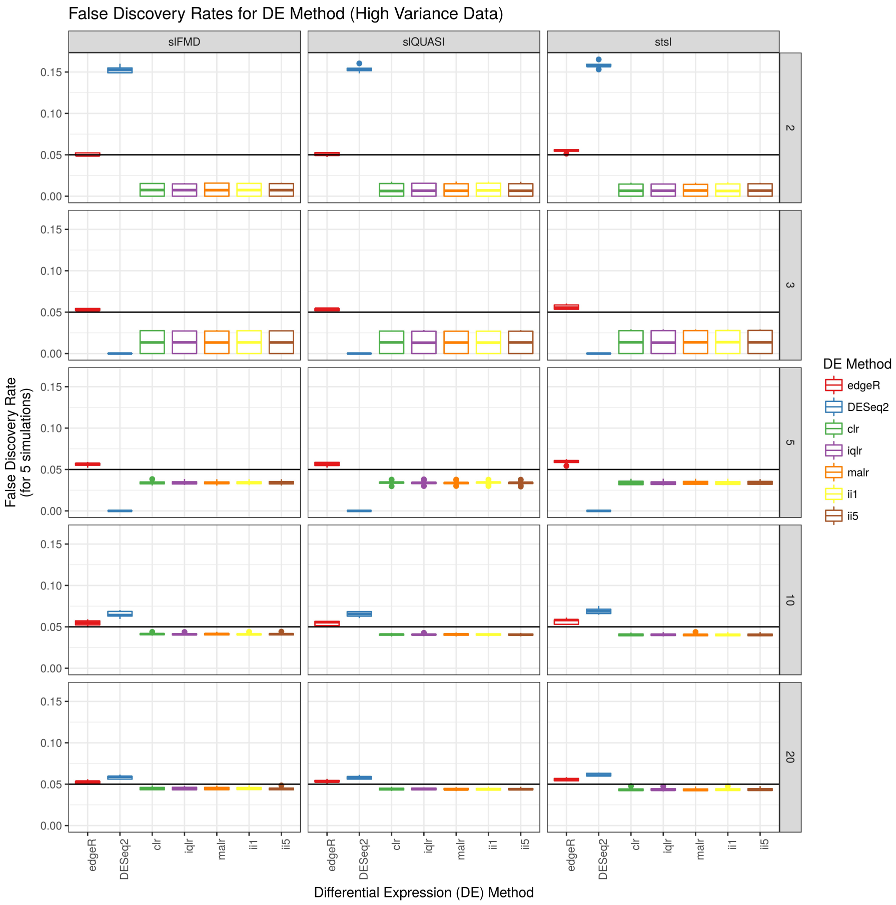

Figure 5: **FDR for the differential expression analysis of high variance data.** This figure shows the false discovery rate (FDR) (y-axis) versus the differential expression method (x-axis). FDR is calculated by analyzing two groups of replicates sampled from the same parent population (repeated 5 times). The results are further organized by alignment and quantification method, and the number of replicates per group. The acronyms clr, iqlr, malr, ii1, and ii5 describe log-ratio transformations (see Methods). The acronyms slFMD, slQUASI, and stsl describe alignment and quantification procedures (see Methods). For these high variance data, edgeR and DESeq2 have an FDR above $\alpha = 0.05$. Of concern, DESeq2 calls 15% of transcripts differentially expressed when there are only two replicates per group and all replicates come from the same population.
