# Supplementary material for: Benchmarking differential expression analysis tools for RNA-Seq: normalization-based vs. log-ratio transformation-based methods
Source: BMC Bioinformatics. 2018 Jul 18;19:274. doi: 10.1186/s12859-018-2261-8 (PMC6052553; doi:10.1186/s12859-018-2261-8)
Supplement: Supplementary file 8 — Analysis scripts. This file contains all the scripts used to generate the simulated data, benchmark methods on the simulated data, benchmark methods on the real data, parse the results, and make the figures. (PDF 3948 kb) [file 12859_2018_2261_MOESM8_ESM.pdf]

Scripts included in this .pdf file:

(01) Calls polyester to generate Wave 1 simulated data
(02) Calls polyester to generate Wave 2 simulated data
(03) Runs tximport on Williams et al. data
(04) Defines functions used to analyze simulated data
(05) Benchmarks performance on simulated samples
(06) Calculates FDR on simulated samples
(07) Benchmarks performance on Williams et al. data
(08) Analyze results and make figures from simulated runs
(09) Analyze results and make figures from FDR runs
(10) Analyze results and make figures from Williams et al. runs
(11) Analyze DE overlap in Rollins et al. data
(12) Analyze DE missed by edgeR

```r
# Note: use branch of kcha/polyester with naive doMC support -- install via:
#  devtools::install_github("kcha/polyester", ref =
"545e33c9776db2927f9a22c8c2f5bfde2b3081a7")
library(polyester)
library(Biostrings)
library(doMC)

# fa = "/home/richardm/bin/STAR-2.5.2/genomes/
Homo_sapiens.GRCh37.dna.primary_assembly.fa"
# gtf = "/home/richardm/bin/STAR-2.5.2/genomes/Homo_sapiens.GRCh37.87.gtf"
# Note: .fa file built using gffread with $fa and $gtf as input, i.e.,
#  gffread $gtf -g $fa gffread-GRCh37.87.fasta
#  mv gffread-GRCh37.87.fasta gffread-GRCh37.87.fa
fasta = "/home/richardm/bin/STAR-2.5.2/genomes/gffread-GRCh37.87.fa"
f <- readDNAStringSet(fasta)
out <- 'xstringset.fa'
writeXStringSet(f, out)

set.seed(3220)
case <- sample(c(4/1, 2/1, 3/2, 1/1, 2/3, 1/2, 1/4),
               size = length(f),
               replace = TRUE,
               prob = c(.03, .07, .09, .7, .06, .03, .02)
)

fold_changes <- as.matrix(data.frame(case, "control" = 1))
colnames(fold_changes) <- NULL

set.seed(3220)

lib_sizes <- c(rnorm(20, mean = 1.1, sd = .05),
               rnorm(20, mean = 0.9, sd = .05)
)

# From polyester vignette,
#  ~20x coverage "here all transcripts will have ~equal FPKM"
readspertx <- round(20 * width(f) / 100)

simulate_experiment(
  'xstringset.fa',
  fold_changes = fold_changes,
  num_reps = c(20, 20),
  fraglen = 300,
  error_model = "illumina4",
  reads_per_transcript = readspertx,
  # meanmodel = TRUE,
  lib_sizes = lib_sizes,
  seed = 3220,

  #size = # use default NULL argument
  outdir = "lo-var",
  cores = 18
)

simulate_experiment(
  'xstringset.fa',
  fold_changes = fold_changes,
  num_reps = c(20, 20),
  fraglen = 300,
  error_model = "illumina4",
  reads_per_transcript = readspertx,
  # meanmodel = TRUE,
  lib_sizes = lib_sizes,
  seed = 3220,

  size = 1, # alternative high-variance
  outdir = "hi-var",
  cores = 18
)
```

```r
# Note: use branch of kcha/polyester with naive doMC support -- install via:
#  devtools::install_github("kcha/polyester", ref =
"545e33c9776db2927f9a22c8c2f5bfde2b3081a7")
library(polyester)
library(Biostrings)
library(foreach)
library(doMC)

# fa = "/home/richardm/bin/STAR-2.5.2/genomes/
Homo_sapiens.GRCh37.dna.primary_assembly.fa"
# gtf = '/home/richardm/bin/STAR-2.5.2/genomes/Homo_sapiens.GRCh37.87.gtf"
# Note: .fa file built using gffread with $fa and $gtf as input, i.e.,
#  gffread $gtf -g $fa gffread-GRCh37.87.fasta
#  mv gffread-GRCh37.87.fasta gffread-GRCh37.87.fa
fasta = '/home/richardm/bin/STAR-2.5.2/genomes/gffread-GRCh37.87.fa"
f <- readDNAStringSet(fasta)
out <- 'xstringset.fa'
writeXStringSet(f, out)

set.seed(3220)
case <- sample(c(4/1, 2/1, 3/2, 1/1, 2/3, 1/2, 1/4),
               size = length(f),
               replace = TRUE,
               prob = c(.03, .07, .09, .7, .06, .03, .02)
)

fold_changes <- as.matrix(data.frame(case, "control" = 1))
colnames(fold_changes) <- NULL

set.seed(6440)
lib_sizes <- c(rnorm(60, mean = 1.1, sd = .05),
               rnorm(60, mean = 0.9, sd = .05)
)

# From polyester vignette,
#  ~20x coverage "here all transcripts will have ~equal FPKM"
readspertx <- round(20 * width(f) / 100)

simulate_experiment(
  'xstringset.fa',
  fold_changes = fold_changes,
  num_reps = c(60, 60),
  fraglen = 300,
  error_model = "illumina4",
  reads_per_transcript = readspertx,
  # meanmodel = TRUE,
  lib_sizes = lib_sizes,
  seed = 6440,

  #size = # use default NULL argument
  outdir = "lo-var",
  cores = 15
)

simulate_experiment(
  'xstringset.fa',
  fold_changes = fold_changes,
  num_reps = c(60, 60),
  fraglen = 300,
  error_model = "illumina4",
  reads_per_transcript = readspertx,
  # meanmodel = TRUE,
  lib_sizes = lib_sizes,
  seed = 6440,

  size = 1, # alternative high-variance
  outdir = "hi-var",
  cores = 15
)
```

```r
library(tximport)
library(EnsDb.Hsapiens.v86)
txdb <- EnsDb.Hsapiens.v86
k <- keys(txdb, keytype = "GENEID")
df <- select(txdb, keys = k, keytype = "GENEID", columns = "TXNAME")
tx2gene <- df[, 2:1]  # tx ID, then gene ID

for(i in c("slFMD", "slQuasi", "starsl")){

  print(i)
  files <- list.files(path = i, full.names = TRUE, recursive = TRUE)
  counts.gene <- tximport(files, type = "salmon", tx2gene = tx2gene)
  write.csv(counts.gene, paste0(i, "-tximport.csv"))
}
```

```
#' @param counts A data.frame. The count matrix.
#' @param group A character vector. The labels for each group.
#' @param dir A character vector. The directory in which to save the output.
#' @param txdata A data.frame. The unfiltered truth set provided by polyester.
pipeline.benchmarkDE <- function(counts, group, dir, txdata){

  setwd(dir)

  ### FILTER COUNTS OR NOT
  #
  lapply(list("filter", "nofilter"),
         function(filt){

           if(filt == "filter"){

             keep <- apply(counts, 1, function(x){ sum(x > 10) > ncol(counts)/2
&& !(any(x == 0))})
             write.csv(txdata[rownames(counts[keep, ]), ], "truth-filter.csv")
             ct <- ceiling(as.data.frame(counts[keep, ]))

           }else if(filt == "nofilter"){

             write.csv(txdata[rownames(counts), ], "truth-nofilter.csv")
             ct <- ceiling(as.data.frame(counts))
             ct[ct == 0] <- 1
           }

           ### RUN ALDEx2 ANALYSIS
           #
           lapply(list("malr", "iqlr", "ii1", "ii5", "all"), function(method){

             lapply(list(8, 128), function(mc){

               if(method == "malr"){
                 malr <- which(txdata[rownames(ct),]$foldchange.1 == 1)
                 tt <- ALDEx2::aldex(ct, group, denom = malr, test = "t",
mc.samples = mc)
               }else if(method == "ii1"){
                 tt <- ALDEx2::aldex(ct, group, denom = "iqlr", test = "t",
mc.samples = mc)
                 for(i in 1:1){
                   nonDE.i <- which(rownames(ct) %in% rownames(tt[tt$wi.eBH > .05,
]))
                   tt <- ALDEx2::aldex(ct, group, denom = nonDE.i, test = "t",
mc.samples = mc)
                 }
               }else if(method == "ii5"){
                 tt <- ALDEx2::aldex(ct, group, denom = "iqlr", test = "t",
mc.samples = mc)
                 for(i in 1:5){
                   nonDE.i <- which(rownames(ct) %in% rownames(tt[tt$wi.eBH > .05,
]))
                   tt <- ALDEx2::aldex(ct, group, denom = nonDE.i, test = "t",
mc.samples = mc)
                 }
               }else{
                 tt <- ALDEx2::aldex(ct, group, denom = method, test = "t",
mc.samples = mc)
               }

               write.csv(tt, file = paste0(filt, "-", method, "-", mc, "-tt.csv"))

               # Save ALDEx2 MA plot
               #png(paste0(filt, "-", method, "-", mc, "-MA-05.png"))
               #ALDEx2::aldex.plot(tt, type = "MA", cutoff = .05)
               #dev.off()
             })
           })
           #
```

```
          ###

          ### RUN edgeR ANALYSIS
          #
          y <- edgeR::DGEList(counts = ct, group = group)
          y <- edgeR::calcNormFactors(y)
          y <- edgeR::estimateCommonDisp(y)
          y <- edgeR::estimateTagwiseDisp(y)
          et <- edgeR::exactTest(y)
          tt <- as.data.frame(edgeR::topTags(et, n = nrow(et)))
          write.csv(tt, file = paste0(filt, "-edgeR-tt.csv"))

          # Save edgeR MA plot
          #deGenes <- rownames(tt)[tt$FDR < .05]
          #png(paste0(filt, "-edgeR-MA-05.png"))
          #edgeR::plotSmear(et, de.tags = deGenes, cex = 0.5)
          #dev.off()
          #
          ###

          ### RUN DESeq2 ANALYSIS
          #
          frame <- data.frame(group, row.names = colnames(ct))
          dds <- DESeq2::DESeqDataSetFromMatrix(countData = ct, colData = frame,
design = ~ group)
          dds <- DESeq2::DESeq(dds)
          tt <- as.data.frame(DESeq2::results(dds))
          tt$padj[is.na(tt$padj)] <- 1
          write.csv(tt, file = paste0(filt, "-DESeq2-tt.csv"))

          # Save DESeq2 MA plot
          #png(paste0(filt, "-DESeq2-MA-05.png"))
          #DESeq2::plotMA(dds, alpha = 0.05)
          #dev.off()
          #
          ###
        })
  #
  ###
}

#' @param dir A character vector. The directory in which to save the output.
pipeline.summarizeDE <- function(dir){

  setwd(dir)

  # Load No-Filter and Filter truth sets
  truth.nofilter <- read.csv("truth-nofilter.csv", row.names = 1)
  truth.filter <- read.csv("truth-filter.csv", row.names = 1)

  files <- list.files(pattern = "*tt")
  observed <- lapply(files, function(f) read.csv(f, row.names = 1))
  names(observed) <- files

  s <- lapply(files, function(f){

    ### SORT RESULTS BASED ON TRUTH SET
    #
    res <- observed[[f]]
    if(grepl("nofilter", f)){ truth <- truth.nofilter
    }else{ truth <- truth.filter }
    res <- res[rownames(truth),]

    if(grepl("edgeR", f)){ padj <- "FDR"
    }else if(grepl("DESeq2", f)){ padj <- "padj"
    }else{ padj <- c("we.eBH", "wi.eBH") }
    #
    ###
```

```
    ### TRY TO CALCULATE SENSITIVITY AND SPECIFICITY
    #
    lapply(padj, function(m){

      obs <- rownames(res)[res[, m] < .05]
      all <- rownames(truth)
      tru <- rownames(truth)[truth$DEstatus.1]

      x <- all %in% obs
      y <- all %in% tru

      if(sum(x) == 0){

        precision <- NA
        recall <- NA

      }else{

        conf <- table(x, y)
        precision <- conf["TRUE", "TRUE"] / (conf["TRUE", "FALSE"] + conf["TRUE",
"TRUE"])
        recall <- conf["TRUE", "TRUE"] / (conf["FALSE", "TRUE"] + conf["TRUE",
"TRUE"])
      }

      data.frame("b" = basename(getwd()), "f" = f, "m" = m,
                 "p" = precision, "r" = recall,
                 "all.obs" = nrow(res),
                 "all.tru" = nrow(truth),
                 "obs" = sum(x),
                 "tru" = sum(y))
    })
    #
    ###
  })

  s <- do.call("rbind", lapply(s, function(o) do.call("rbind", o)))
  write.csv(s, "summary.csv")
  return(s)
}

#' @param dir A character vector. The directory in which to save the output.
pipeline.summarizeFDR <- function(dir){

  setwd(dir)

  files <- list.files(pattern = "*tt")
  observed <- lapply(files, function(f) read.csv(f, row.names = 1))
  names(observed) <- files

  s <- lapply(files, function(f){

    ### SET P-VALUE COLUMN(S)
    #
    res <- observed[[f]]

    if(grepl("edgeR", f)){ padj <- "PValue"
    }else if(grepl("DESeq2", f)){ padj <- "pvalue"
    }else{ padj <- c("we.ep", "wi.ep") }
    #
    ###

    ### CALCULATE FDR
    #
    lapply(padj, function(m){

      obs <- sum(res[, m] < .05)
      all <- nrow(res)
```

```
        data.frame("b" = basename(getwd()), "f" = f, "m" = m,
                   "obs" = obs, "all" = all, "fdr" = obs/all)
      })
      #
      ###
    })

    s <- do.call("rbind", lapply(s, function(o) do.call("rbind", o)))
    write.csv(s, 'summary.csv')
    return(s)
  }
```

```r
################################################################
### INSTALL THESE PACKAGES AND SET WORKING DIRECTORY

#ALDEx2 -- from Bioconductor
#edgeR -- from Bioconductor
#DESeq2 -- from Bioconductor
#testthat
#propr
#plyr
#doMC
library(doMC)
registerDoMC(cores = 30)
wd <- "/home/quinnt/rds-collection-mount/Analysis-Benchmark/bench-simulated-rev/"
`%+%` <- function(a, b) paste0(a, b)
source(wd %+% "1-bench-pipeline.R")
NFEATS <- 10000
NBOOTS <- 20

################################################################
### DELETE THESE AFTER DE-BUGGING

#registerDoMC(cores = 2)
#wd <- "/home/thom/Downloads/bench-simulated-rev/"
#source(wd %+% "1-bench-pipeline.R")
#NFEATS <- 100
#NBOOTS <- 3

################################################################
### RUN THE FOLLOWING AS IS -- IMPORT DATA

setwd(wd)

groupdata <- read.table(wd %+% "sim_info/sim_rep_info.txt", stringsAsFactors =
FALSE)
groupdata$rep_id <- make.names(groupdata$rep_id, unique = TRUE)
group1 <- which(groupdata$group == 1)
group2 <- which(groupdata$group == 2)

txdata <- read.table("sim_info/sim_tx_info.txt", stringsAsFactors = FALSE)
rows.clean <- unlist(lapply(strsplit(txdata[,1], "\\s"), function(x) x[1]))
rownames(txdata) <- rows.clean

# Step through transcript-level files (i.e., not stst)
files <- list.files("sim_counts", full.names = TRUE)
index.sim <- grepl("sim_", basename(files)) & !grepl("stst_", basename(files))
files <- files[index.sim]

# Force equivalent row order
counts <- lapply(files, function(x) read.csv(x, row.names = 1))
all(rownames(counts[[1]]) %in% rownames(txdata))
counts <- lapply(counts, function(x) x[rownames(txdata), ])
names(counts) <- gsub(".csv", "", basename(files))

for(i in 1:length(counts)){
  colnames(counts[[i]]) <- gsub("_quant.sf", "", colnames(counts[[i]]))
  if(!all(colnames(counts[[i]]) %in% groupdata$rep_id)) stop()
  counts[[i]] <- counts[[i]][, groupdata$rep_id]
}

for(i in 1:length(counts)){
  for(j in 1:length(counts)){
    testthat::expect_equal(
      nrow(counts[[i]]),
      nrow(counts[[j]])
    )
  }
}

for(i in 1:length(counts)){
```

```
  for(j in 1:length(counts)){
    testthat::expect_equal(
      colnames(counts[[i]]),
      colnames(counts[[j]])
    )
  }
}

for(i in 1:length(counts)){
  for(j in 1:length(counts)){
    testthat::expect_equal(
      rownames(counts[[i]]),
      rownames(counts[[j]])
    )
  }
}

###############################################################
### RUN THE FOLLOWING AS IS -- BENCHMARK METHODS

grid <- expand.grid("file" = gsub(".csv", "", basename(files)),
                    "N" = c(2, 3, 5, 10, 20),
                    stringsAsFactors = FALSE)
foreach(i = 1:nrow(grid)) %dopar% {

  # Set directory to bench-simulated-rev/file/N
  setwd(wd)
  system("mkdir " %+% grid[i, "file"])
  dir <- paste(grid[i,], collapse = "/")
  system("mkdir " %+% dir)
  setwd(wd %+% dir)

  p <- lapply(1:NBOOTS, function(b){

    # Set directory to bench-simulated-rev/file/N/b
    setwd(wd %+% dir)
    system("mkdir " %+% b)
    setwd(wd %+% dir %+% "/" %+% b)

    f <- grid[i, "file"]
    n <- grid[i, "N"]

    set.seed(b)
    sample.tx <- sample(rownames(txdata), NFEATS) # character
    sample.g1 <- sample(group1, n) # numeric
    sample.g2 <- sample(group2, n) # numeric

    counts.b <- counts[[f]][sample.tx, c(sample.g1, sample.g2)]
    group.b <- c(rep("A", n), rep("B", n))

    pipeline.benchmarkDE(counts.b, group.b, getwd(), txdata)
    pipeline.summarizeDE(getwd())
  })

  setwd(wd %+% dir)
  p <- do.call("rbind", p)
  p <- suppressWarnings(cbind(grid[i,,], p))
  write.csv(p, file = "performances.csv")
}

setwd(wd)
p <- list.files(pattern = "performances.csv", recursive = TRUE, full.names = TRUE)
p <- lapply(p, read.csv)
p <- do.call("rbind", p)
write.csv(p, "bench-simulated-rev-OUT.csv")
```

```
#################################################################
### INSTALL THESE PACKAGES AND SET WORKING DIRECTORY

#ALDEx2 -- from Bioconductor
#edgeR -- from Bioconductor
#DESeq2 -- from Bioconductor
#testthat
#propr
#plyr
#doMC
library(doMC)
registerDoMC(cores = 8)
wd <- "/home/quinnt/rds-collection-mount/Analysis-Benchmark/bench-simulated-fdr/"
`%+%` <- function(a, b) paste0(a, b)
source(wd %+% "1-bench-pipeline.R")
NFEATS <- 10000
NBOOTS <- 5

#################################################################
### DELETE THESE AFTER DE-BUGGING

#registerDoMC(cores = 2)
#wd <- "/home/thom/Downloads/bench-simulated-fdr/"
#source(wd %+% "1-bench-pipeline.R")
#NFEATS <- 100
#NBOOTS <- 3

#################################################################
### RUN THE FOLLOWING AS IS -- IMPORT DATA

setwd(wd)

groupdata <- read.table(wd %+% "sim_info/sim_rep_info.txt", stringsAsFactors =
FALSE)
groupdata$rep_id <- make.names(groupdata$rep_id, unique = TRUE)
group1 <- which(groupdata$group == 1)
group2 <- which(groupdata$group == 2)

txdata <- read.table("sim_info/sim_tx_info.txt", stringsAsFactors = FALSE)
rows.clean <- unlist(lapply(strsplit(txdata[,1], "\\s"), function(x) x[1]))
rownames(txdata) <- rows.clean

# Step through transcript-level files (i.e., not stst)
files <- list.files("sim_counts", full.names = TRUE)
index.sim <- grepl("sim_", basename(files)) & !grepl("stst_", basename(files))
files <- files[index.sim]

# Force equivalent row order
counts <- lapply(files, function(x) read.csv(x, row.names = 1))
all(rownames(counts[[1]]) %in% rownames(txdata))
counts <- lapply(counts, function(x) x[rownames(txdata), ])
names(counts) <- gsub(".csv", "", basename(files))

for(i in 1:length(counts)){
  colnames(counts[[i]]) <- gsub("_quant.sf", "", colnames(counts[[i]]))
  if(!all(colnames(counts[[i]]) %in% groupdata$rep_id)) stop()
  counts[[i]] <- counts[[i]][, groupdata$rep_id]
}

for(i in 1:length(counts)){
  for(j in 1:length(counts)){
    testthat::expect_equal(
      nrow(counts[[i]]),
      nrow(counts[[j]])
    )
  }
}

for(i in 1:length(counts)){
```

```
  for(j in 1:length(counts)){
    testthat::expect_equal(
      colnames(counts[[i]]),
      colnames(counts[[j]])
    )
  }
}

for(i in 1:length(counts)){
  for(j in 1:length(counts)){
    testthat::expect_equal(
      rownames(counts[[i]]),
      rownames(counts[[j]])
    )
  }
}

###############################################################
### RUN THE FOLLOWING AS IS -- BENCHMARK METHODS

grid <- expand.grid("file" = gsub(".csv", "", basename(files)),
                    "N" = c(2, 3, 5, 10, 20),
                    stringsAsFactors = FALSE)
foreach(i = 1:nrow(grid)) %dopar% {

  # Set directory to bench-simulated-rev/file/N
  setwd(wd)
  system("mkdir " %+% grid[i, "file"])
  dir <- paste(grid[i,], collapse = "/")
  system("mkdir " %+% dir)
  setwd(wd %+% dir)

  p <- lapply(1:NBOOTS, function(b){

    # Set directory to bench-simulated-rev/file/N/b
    setwd(wd %+% dir)
    system("mkdir " %+% b)
    setwd(wd %+% dir %+% '/' %+% b)

    f <- grid[i, "file"]
    n <- grid[i, "N"]

    # ALL SAMPLES COME FROM SAME GROUP
    set.seed(b)
    sample.tx <- sample(rownames(txdata), NFEATS) # character
    sample.g1 <- sample(group1, n * 2) # numeric
    #sample.g2 <- sample(group2, n) # numeric

    counts.b <- counts[[f]][sample.tx, c(sample.g1)]
    group.b <- c(rep("A", n), rep("B", n))

    pipeline.benchmarkDE(counts.b, group.b, getwd(), txdata)
    pipeline.summarizeFDR(getwd())
  })

  setwd(wd %+% dir)
  p <- do.call("rbind", p)
  p <- suppressWarnings(cbind(grid[i,], p))
  write.csv(p, file = "performances.csv")
}

setwd(wd)
p <- list.files(pattern = "performances.csv", recursive = TRUE, full.names = TRUE)
p <- lapply(p, read.csv)
p <- do.call("rbind", p)
write.csv(p, "bench-simulated-fdr-OUT.csv")
```

```r
###############################################################
### LOCATION OF FILE DEPENDENCIES

library(doMC)
registerDoMC(cores = 5) # only need 5 cores

# Working directory containg "counts/gene" and "counts/tx" folders:
wd <- "/home/quinnt/rds-collection-mount/Analysis-Benchmark/bench-williams-rev/"

# Conversion table (from Williams 2017):
convertTable <- "conversion_tgs.txt"

# Microarray reference (from Williams 2017):
microarrayReference <- "12859_2016_1457_MOESM2_ESM.xlsx"

# Use meta-data to assign group labels (from Williams 2017):
meta <- read.delim("bench-williams-meta.txt")
grp <- substr(as.character(meta$Library_Name_s), 1, 3)

# Figure table (from Williams 2017):
fig.file <- "12859_2016_1457_MOESM6_ESM.xlsx"

###############################################################
### FUNCTIONS USED FOR BENCHMARKING

#' Read Gene Expression Data Files
#'
#' This function (a) reads in gene expression data files and
#'  (b) ensures equivalent row and column names.
#'
#' NOTE: Remove 1:5000 subset later
getCounts <- function(files){

  # Read files into R
  counts <- lapply(files, function(x) read.csv(x, row.names = 1))

  # Special handling for tximport data
  counts <- lapply(counts, function(x){
    if(any(grepl("counts\\.", colnames(x)))){
      x[, grepl("counts\\.", colnames(x))]
    }else{
      x
    }
  })

  # Ensure all data sets have the same feature names
  feats.int <- rownames(counts[[1]])
  for(i in 1:length(counts)){
    feats.int <- intersect(feats.int, rownames(counts[[i]]))
  }
  counts <- lapply(counts, function(x) x[feats.int, ])

  # # Remove later
  #set.seed(1)
  #short <- sample(nrow(counts[[1]]))[1:5000]
  #counts <- lapply(counts, function(x) x[short,])

  for(i in 1:length(counts)){
    for(j in 1:length(counts)){
      testthat::expect_equal(
        nrow(counts[[i]]),
        nrow(counts[[j]])
      )
    }
  }

  for(i in 1:length(counts)){
    for(j in 1:length(counts)){
      testthat::expect_equal(
```

```
        ncol(counts[[i]]),
        ncol(counts[[j]])
      )
    }
  }

  for(i in 1:length(counts)){
    for(j in 1:length(counts)){
      testthat::expect_equal(
        rownames(counts[[i]]),
        rownames(counts[[j]])
      )
    }
  }

  return(counts)
}

#' Run edgeR as per Williams 2017
edgeR.williams <- function(expMat, group, method){

  library(edgeR)

  y<-DGEList(counts=expMat,group=group)
  y<-calcNormFactors(y)

  # Filter for counts present in half the samples, on cpm data
  cutoff<-ncol(expMat)/2
  keep <- rowSums(cpm(y)>1) >= cutoff
  y <- y[keep, , keep.lib.sizes=FALSE]

  ## Recalculate norm factors after filtering
  y<-calcNormFactors(y)

  y<-estimateCommonDisp(y)
  y<-estimateTagwiseDisp(y)
  et<-exactTest(y)
  adjp<-topTags(et,n=nrow(y))

  return(adjp)
}

#' Run DESeq2 as per Williams 2017
DESeq2.williams <- function(expMat, group, method){

  # DESeq2 requires integer input
  expMat <- ceiling(as.data.frame(expMat))

  frame <- data.frame(group, row.names = colnames(expMat))
  dds <- DESeq2::DESeqDataSetFromMatrix(countData = expMat, colData = frame,
design = ~ group)
  dds <- DESeq2::DESeq(dds)
  tt <- as.data.frame(DESeq2::results(dds))
  tt$padj[is.na(tt$padj)] <- 1

  colnames(tt)[colnames(tt) == "padj"] <- "FDR"

  return(tt)
}

#' Run ALDEx2
#'
#' Uses 'wi.eBH' as the "FDR" column
ALDEx2.williams <- function(ct, group, method){

  ct[ct == 0] <- 1
  ct <- ceiling(as.data.frame(ct))

  if(method == "iil"){
```

```r
    tt <- ALDEx2::aldex(ct, group, denom = "iqlr", test = "t", mc.samples = 128,
effect = FALSE)
    for(i in 1:1){
      nonDE.i <- which(rownames(ct) %in% rownames(tt[tt$wi.eBH > .05, ]))
      tt <- ALDEx2::aldex(ct, group, denom = nonDE.i, test = "t", mc.samples =
128, effect = FALSE)
    }
  }else if(method == "ii5"){
    tt <- ALDEx2::aldex(ct, group, denom = "iqlr", test = "t", mc.samples = 128,
effect = FALSE)
    for(i in 1:5){
      nonDE.i <- which(rownames(ct) %in% rownames(tt[tt$wi.eBH > .05, ]))
      tt <- ALDEx2::aldex(ct, group, denom = nonDE.i, test = "t", mc.samples =
128, effect = FALSE)
    }
  }else{
    tt <- ALDEx2::aldex(ct, group, denom = method, test = "t", mc.samples = 128,
effect = FALSE)
  }

  # Use wi.eBH column for FDR
  #ALDEx2::aldex.plot(tt, type = "MW", cutoff = .05)
  colnames(tt)[colnames(tt) == "wi.eBH"] <- "FDR"

  return(tt)
}

#' Convert ENSG and/or ENST to Symbol
#'
#' Uses 'conversionTable' as provided by cckim47.
convertToSymbol <- function(adjp, conversionTable){

  if(all(grepl('ENSG', rownames(adjp)))){
    compType <- "gene"
    message("Converting gene ID to SYMBOL.")
  }else{
    compType <- "tx"
    message("Converting transcript ID to SYMBOL.")
  }

  ## Merge gene name information into results
  if(compType == "gene"){
    resSymbol<-merge(adjp,conversionTable[,c("ENSG","geneName")],
by.x="row.names", by.y="ENSG")
    colnames(resSymbol) = c("ENSG",colnames(resSymbol[2:length(resSymbol)]))
  } else{
    resSymbol<-merge(adjp,conversionTable, by.x="row.names", by.y="ENST")
    colnames(resSymbol) = c("ENST",colnames(resSymbol[2:length(resSymbol)]))
  }

  return(resSymbol)
}

#' Get Gene Universe for Microarray Reference
#'
#' We must filter gene symbols through the intersection
#'  of the RNA-Seq and microarray data sets. This changes
#'  for each microarray reference!
getUniverse <- function(id, rnaseqset){

  library(AnnotationDbi)

  # lapply(c("int-Frank", "intHaniffa", "int-Ingersoll", "int-Wong")
  if(id == 1){ # int-Frank

    message("int-Frank")
    # GPL570    [HG-U133_Plus_2] Affymetrix Human Genome U133 Plus 2.0 Array
    library(hgu133plus2.db)
    db <- hgu133plus2.db
```

```r
  }else if(id == 2){ # int-Haniffa

    message("int-Haniffa")
    # GPL10558  Illumina HumanHT-12 V4.0 expression beadchip
    library(illuminaHumanv4.db)
    db <- illuminaHumanv4.db

  }else if(id == 3){ # int-Ingersoll

    message("int-Ingersoll")
    # GPL570    [HG-U133_Plus_2] Affymetrix Human Genome U133 Plus 2.0 Array
    library(hgu133plus2.db)
    db <- hgu133plus2.db

  }else if(id == 4){ # int-Wong

    message("int-Wong")
    # GPL6102   Illumina human-6 v2.0 expression beadchip
    library(illuminaHumanv2.db)
    db <- illuminaHumanv2.db
  }

  x <- select(db, keys = keys(db, "PROBEID"), column = "SYMBOL", keytype =
"PROBEID")
  microarrayset <- unique(x[!is.na(x$SYMBOL), "SYMBOL"])
  message("Microarray contains:", length(unique(microarrayset)))
  message("RNA-Seq contains:", length(unique(rnaseqset)))
  i <- intersect(microarrayset, rnaseqset)
  message("Intersection:", length(unique(i)))

  return(i)
}

pipeline <- function(counts, grp, func, method, title){

  # (1) Run DE analyses
  de <- lapply(counts, func, group = grp, method = method)

  # (2) Use Conversion table to Convert to common names
  conversionTable <- read.table(convertTable, header = TRUE)
  de.symbol <- lapply(de, convertToSymbol, conversionTable = conversionTable)

  # (3) Pull in microarray reference (i.e., genes selected as significant)
  sheets <- c("int-Frank", "int-Haniffa", "int-Ingersoll", "int-Wong")
  intersects <- lapply(sheets,
                       function(s) as.data.frame(
                         readxl::read_excel(microarrayReference, sheet = s))[,1])

  # (4) Precision and recall -- for each microarray reference
  # (4.a) Filter microarray reference and RNA-Seq
  # "we first filtered each reference and
  # RNA-Seq gene set to include only features measurable
  # both by RNA-Seq (i.e., present in the GRCh37 genome
  # release) and by the microarray (i.e., a probe targeting the
  # feature was present on the microarray platform) within a
  # given comparison"
  out <- lapply(de.symbol, function(de){

    # For each RNA-Seq data set, move through each microarray data set...
    lapply(1:length(intersects), function(i){

      # MUST FILTER BEFORE PULLING OUT SIGNIFICANT SYMBOLS
      # Get i-th microarray gene universe
      UNIONSET <- getUniverse(i, rnaseqset = de[, "geneName"])

      # PULL OUT SIGNIFICANT SYMBOLS
      sigSymbols <- function(x, col.fdr = "FDR", col.symbol = "geneName"){
        as.character(unique(x[x[, col.fdr] < .05, col.symbol]))
```

```
    }
    de.fdr <- sigSymbols(de, col.fdr = "FDR", col.symbol = "geneName")
    message("Total probes:", nrow(de))
    message("FDR probes:", length(de.fdr))

    ref <- intersects[[i]]
    l <- list(
      "de.fdr" = de.fdr[de.fdr %in% UNIONSET],
      "ref" = ref[ref %in% UNIONSET]
    )
  })
})

# (4.b) Calculate precision and recall for each microarray reference
# "Recall was calculated as the number of signifi-
# cant genes in the intersection of the test RNA-Seq dataset
# with the reference dataset, divided by the number of genes
# identified as significant in the reference dataset."
# "Precision was calculated as the number of significant genes in the
# intersection of the test RNA-Seq dataset with the refer-
# ence dataset, divided by the number of genes identified as
# significant in the test RNA-Seq dataset."
precision <-
  lapply(out,
         function(o) sapply(o, function(x) length(intersect(x[["de.fdr"]], x
[["ref"]])) / length(x[["de.fdr"]])))
recall <-
  lapply(out,
         function(o) sapply(o, function(x) length(intersect(x[["de.fdr"]], x
[["ref"]])) / length(x[["ref"]])))

# (4.c) Average precision and recall
# "Here, we have depicted
# our results using performance metrics averaged across all
# four references;"
precision.mean <- lapply(precision, mean)
recall.mean <- lapply(recall, mean)

# Compile data
data.frame(
  file = c(files.gene, files.tx),
  DE = title,
  type = sapply(counts, function(x) ifelse(grepl("ENSG", rownames(x)[1]),
"gene", "tx")),
  FrankPrecision = sapply(precision, function(x) x[1]),
  HaniffaPrecision = sapply(precision, function(x) x[2]),
  IngersolPrecisionl = sapply(precision, function(x) x[3]),
  WongPrecision = sapply(precision, function(x) x[4]),
  AveragePrecision = unlist(precision.mean),
  FrankRecall = sapply(recall, function(x) x[1]),
  HaniffaRecall = sapply(recall, function(x) x[2]),
  IngersollRecall = sapply(recall, function(x) x[3]),
  WongRecall = sapply(recall, function(x) x[4]),
  AverageRecall = unlist(recall.mean)
)
}

###############################################################
### RUN THIS AS-IS

files.gene <- list.files("counts/gene", full.names = TRUE)
files.tx <- list.files("counts/tx", full.names = TRUE)
counts <- c(getCounts(files.gene), getCounts(files.tx))

foreach(run = 1:5) %dopar% {
  # if(run == 1){
  #
  #   out.edgeR <- pipeline(counts, grp, edgeR.williams, title = "edgeR")
  #   write.csv(out.edgeR, "bench-edgeR.csv")
```

```
  # }
  # if(run == 2){
  #
  #   out.clr <- pipeline(counts, grp, ALDEx2.williams, title = "clr", method =
"clr")
  #   write.csv(out.clr, "bench-clr.csv")
  # }
  # if(run == 3){
  #
  #   out.iqlr <- pipeline(counts, grp, ALDEx2.williams, title = "iqlr", method =
"iqlr")
  #   write.csv(out.iqlr, "bench-iqlr.csv")
  # }
  # if(run == 4){
  #
  #   out.ii1 <- pipeline(counts, grp, ALDEx2.williams, title = "ii1", method =
"ii1")
  #   write.csv(out.ii1, "bench-ii1.csv")
  # }
  if(run == 5){

    out.DESeq2 <- pipeline(counts, grp, DESeq2.williams, title = "DESeq2")
    write.csv(out.DESeq2, "bench-DESeq2.csv")
  }
}
```

```
df <- read.csv(
  "/home/thom/Dropbox/R/projects/manuscripts/benchmark/1-sims/bench-simulated-rev-
OUT.csv",
  stringsAsFactors = FALSE
)

df$filter <- unlist(lapply(strsplit(df$f, "-"), function(x) x[1]))
df$method <- unlist(lapply(strsplit(df$f, "-"), function(x) x[2]))
df$method <- ifelse(df$method == "all", "clr", df$method)
df$method <-
  factor(df$method,
         levels = c("edgeR", "DESeq2", "clr", "iqlr", "malr", "ii1", "ii5"))
df$mc <- unlist(lapply(strsplit(df$f, "-"), function(x) x[3]))
df$data <- unlist(lapply(strsplit(df$file, "_"), function(x) x[2]))
df$process <- unlist(lapply(strsplit(df$file, "_|(out)"), function(x) x[3]))

# (A) Statistical tests for filter, mc, m, etc.

# across 3000 = 20 B, 5 n, 3 proc, 2 filters, and 5 lrs
t.test(df[df$data == "lv" & df$m == "wi.eBH" & df$mc == 8, "r"],
       df[df$data == "lv" & df$m == "wi.eBH" & df$mc == 128, "r"])
t.test(df[df$data == "lv" & df$m == "wi.eBH" & df$mc == 8, "p"],
       df[df$data == "lv" & df$m == "wi.eBH" & df$mc == 128, "p"])

# across 3000 = 20 B, 5 n, 3 proc, 2 , mcs, and 5 lrs
t.test(df[df$data == "lv" & df$m == "wi.eBH" & df$filter == "filter", "r"],
       df[df$data == "lv" & df$m == "wi.eBH" & df$filter == "nofilter", "r"])
t.test(df[df$data == "lv" & df$m == "wi.eBH" & df$filter == "filter", "p"],
       df[df$data == "lv" & df$m == "wi.eBH" & df$filter == "nofilter", "p"])

# across 300 = 20 B, 5 n, 3 proc
t.test(df[df$data == "lv" & df$method == "edgeR" & df$filter == "filter", "r"],
       df[df$data == "lv" & df$method == "edgeR" & df$filter == "nofilter", "r"])
t.test(df[df$data == "lv" & df$method == "edgeR" & df$filter == "filter", "p"],
       df[df$data == "lv" & df$method == "edgeR" & df$filter == "nofilter", "p"])

# across 300 = 20 B, 5 n, 3 proc
t.test(df[df$data == "lv" & df$method == "DESeq2" & df$filter == "filter", "r"],
       df[df$data == "lv" & df$method == "DESeq2" & df$filter == "nofilter", "r"])
t.test(df[df$data == "lv" & df$method == "DESeq2" & df$filter == "filter", "p"],
       df[df$data == "lv" & df$method == "DESeq2" & df$filter == "nofilter", "p"])

# across 60 = 20 B, 3 proc
lv <- df[df$data == "lv" & df$m != "we.eBH" & df$filter == "nofilter" & df$mc !=
8,]
library(plyr)
pval <- ldply(unique(lv$N), function(n){
  ldply(unique(lv$method), function(m1){
    ldply(unique(lv$method), function(m2){

      if(m1 != m2){

        a <- lv[lv$N == n & lv$method == m1, "p"]
        b <- lv[lv$N == n & lv$method == m2, "p"]
        pp <- tryCatch(t.test(a, b)$p.value, error = function(e) return(NA))

        a <- lv[lv$N == n & lv$method == m1, "r"]
        b <- lv[lv$N == n & lv$method == m2, "r"]
        rp <- tryCatch(t.test(a, b)$p.value, error = function(e) return(NA))

        data.frame(n, m1, m2, pp, rp)
      }
    })
  })
})

# across 210 = 7 methods, 6 methods, 5 n
pval[pval$n == 5,]
pval[pval$n == 10,]
```

```r
pval[pval$n == 20,]

# (B) Pre-process data for plots

df.prec <- df$p
df.reca <- df$r
df <- rbind(df, df)
df$Amount <- c(df.prec, df.reca)
df$Type <- c(rep("Precision", nrow(df)/2), rep("Recall", nrow(df)/2))

# (C) Prepare plot functions

pDEMethod <- function(df, title){
  ggplot(data = df, aes(x = method, y = Amount, color = as.factor(method))) +
    geom_boxplot() + facet_grid(Type ~ N) +
    theme_bw() + ylim(0, 1) +
    xlab("Differential Expression (DE) Method") + ylab("Performance") +
    labs(color = "DE Method") +
    scale_colour_brewer(palette = "Set1") +
    ggtitle(title) +
    geom_hline(data = df[df$Type == "Precision",], aes(yintercept = .95)) +
    theme(axis.text.x = element_text(angle = 90, hjust = 1))
}

pProcess <- function(df, title){
  ggplot(data = df, aes(x = method, y = Amount, color = as.factor(N))) +
    geom_point() + facet_grid(Type ~ process) +
    theme_bw() + ylim(0, 1) +
    xlab("Differential Expression (DE) Method") + ylab("Performance") +
    labs(color = "Sample Size") +
    scale_colour_brewer(palette = "Set1") +
    ggtitle(title) +
    geom_hline(data = df[df$Type == "Precision",], aes(yintercept = .95)) +
    theme(axis.text.x = element_text(angle = 90, hjust = 1))
}

# (D) Plot wi.eBH data

library(ggplot2)

###
# FIGURE 1:
jpeg("/home/thom/Dropbox/R/projects/manuscripts/benchmark/1-sims/fig1.jpg",
     width = 10, height = 10, units = "in", res = 600)
pDEMethod(df[df$m != "we.eBH" & df$mc != 8 & df$filter == "nofilter" & df$data ==
"lv",],
          "Differential Expression Performance using a Simulated Reference (Low
Variance Data)")
dev.off()

###
# FIGURE 2:
jpeg("/home/thom/Dropbox/R/projects/manuscripts/benchmark/1-sims/fig2.jpg",
     width = 10, height = 10, units = "in", res = 600)
pProcess(df[df$m != "we.eBH" & df$mc != 8 & df$filter == "nofilter" & df$data ==
"lv",],
         "Differential Expression Performance using a Simulated Reference (Low
Variance Data)")
dev.off()

###
# FIGURE 3:
jpeg("/home/thom/Dropbox/R/projects/manuscripts/benchmark/1-sims/fig3.jpg",
     width = 10, height = 10, units = "in", res = 600)
pDEMethod(df[df$m != "we.eBH" & df$mc != 8 & df$filter == "nofilter" & df$data ==
"hv",],
          "Differential Expression Performance using a Simulated Reference (High
Variance Data)")
dev.off()
```

```r
# (E) Plot wi.eBH data

###
# SUPP 1:
jpeg("/home/thom/Dropbox/R/projects/manuscripts/benchmark/1-sims/sup1.jpg",
     width = 10, height = 10, units = "in", res = 600)
pDEMethod(df[df$m != "wi.eBH" & df$mc != 8 & df$filter == "nofilter" & df$data ==
"lv",],
          "Differential Expression Performance using a Simulated Reference (Low
Variance Data) [we.eBH column]")
dev.off()

###
# SUPP 2:
jpeg("/home/thom/Dropbox/R/projects/manuscripts/benchmark/1-sims/sup2.jpg",
     width = 10, height = 10, units = "in", res = 600)
pProcess(df[df$m != "wi.eBH" & df$mc != 8 & df$filter == "nofilter" & df$data ==
"lv",],
         "Differential Expression Performance using a Simulated Reference (Low
Variance Data) [we.eBH column]")
dev.off()

###
# SUPP 3:
jpeg("/home/thom/Dropbox/R/projects/manuscripts/benchmark/1-sims/sup3.jpg",
     width = 10, height = 10, units = "in", res = 600)
pDEMethod(df[df$m != "wi.eBH" & df$mc != 8 & df$filter == "nofilter" & df$data ==
"hv",],
          "Differential Expression Performance using a Simulated Reference (High
Variance Data) [we.eBH column]")
dev.off()
```

```
df <- read.csv(
  "/home/thom/Dropbox/R/projects/manuscripts/benchmark/2-fdr/bench-simulated-fdr-
OUT.csv",
  stringsAsFactors = FALSE
)

df$fdr[is.na(df$fdr)] <- 0
df$filter <- unlist(lapply(strsplit(df$f, "-"), function(x) x[1]))
df$method <- unlist(lapply(strsplit(df$f, "-"), function(x) x[2]))
df$method <- ifelse(df$method == "all", "clr", df$method)
df$method <-
  factor(df$method,
         levels = c("edgeR", "DESeq2", "clr", "iqlr", "malr", "ii1", "ii5"))
df$mc <- unlist(lapply(strsplit(df$f, "-"), function(x) x[3]))
df$data <- unlist(lapply(strsplit(df$file, "_"), function(x) x[2]))
df$process <- unlist(lapply(strsplit(df$file, "_|(out)"), function(x) x[3]))
df <- df[df$m != "we.iBH" & df$mc != 8 & df$filter == "nofilter",]

jpeg("/home/thom/Dropbox/R/projects/manuscripts/benchmark/2-fdr/sup4.jpg",
     width = 10, height = 10, units = "in", res = 600)
library(ggplot2)
ggplot(df[df$data == "lv",], aes(x = method, y = fdr, color = method)) +
geom_boxplot() +
  facet_grid(N ~ process) + theme_bw() +
  xlab("Differential Expression (DE) Method") +
  ylab("False Discovery Rate\n(for 5 simulations)") +
  labs(color = "DE Method") +
  scale_colour_brewer(palette = "Set1") +
  ggtitle("False Discovery Rates for DE Method (Low Variance Data)") +
  geom_hline(yintercept = .05) +
  theme(axis.text.x = element_text(angle = 90, hjust = 1))
dev.off()

jpeg("/home/thom/Dropbox/R/projects/manuscripts/benchmark/2-fdr/sup5.jpg",
     width = 10, height = 10, units = "in", res = 600)
library(ggplot2)
ggplot(df[df$data == "hv",], aes(x = method, y = fdr, color = method)) +
geom_boxplot() +
  facet_grid(N ~ process) + theme_bw() +
  xlab("Differential Expression (DE) Method") +
  ylab("False Discovery Rate\n(for 5 simulations)") +
  labs(color = "DE Method") +
  scale_colour_brewer(palette = "Set1") +
  ggtitle("False Discovery Rates for DE Method (High Variance Data)") +
  geom_hline(yintercept = .05) +
  theme(axis.text.x = element_text(angle = 90, hjust = 1))
dev.off()
```

```r
setwd("/home/thom/Dropbox/R/projects/manuscripts/benchmark/3-williams/")
files <- list.files(pattern = "bench.*csv")
f <- lapply(files, read.csv)
will <- do.call("rbind", f)
will <- will[, -1]
colnames(will)[1:3] <- c("InputFile", "DEmethod", "Type")
write.csv(as.matrix(will), file = "will-clean.csv")

# Recreate figure from Williams et al.
#  -- [, c(3, 4)] is gene-wise R/P; [, c(7, 8)] is tx-wise R/P
file <- "/home/thom/Dropbox/R/projects/manuscripts/benchmark/3-
williams/12859_2016_1457_MOESM6_ESM.xlsx"
measures <- lapply(c(3, 4, 7, 8), function(x) readxl::read_excel(file, sheet = x))
fig.data <- plyr::rbind.fill(
  cbind("type" = "gene", merge(measures[[1]], measures[[2]])),
  cbind("type" = "tx", merge(measures[[3]], measures[[4]]))
)
colnames(fig.data) <- gsub("\\s", "", colnames(fig.data))

fig.data <- fig.data[, c("type", "DE", "AveragePrecision", "AverageRecall")]
fig.data$Process <- "Data from \nWilliams et al.\n(2017)"
fig.data$alpha <- .9
fig.data$DE <- "Data from \nWilliams et al.\n(2017)"

new <- read.csv("/home/thom/Dropbox/R/projects/manuscripts/benchmark/3-williams/
will-clean.csv",
                stringsAsFactors = FALSE)
new$Process <- sapply(new$InputFile, function(x) strsplit(x, split = "/|_|-")[[1]]
[3])
new$Process[new$Process == "starsl"] <- "stsl"
new$Process[new$Process == "slQuasi"] <- "slQUASI"
fig.new <- new[, c("Type", "DEmethod", "AveragePrecision", "AverageRecall",
"Process")]
fig.new$alpha <- 1
colnames(fig.new) <- colnames(fig.data)
fig <- rbind(fig.data, fig.new)
fig$DE <-
  factor(fig$DE,
         levels = c("edgeR", "DESeq2", "clr", "iqlr", "malr", "ii1",
                    "Data from \nWilliams et al.\n(2017)"))
fig$type <- ifelse(fig$type == "tx", "Transcript-level", "Gene-level")
fig$Process <-
  factor(fig$Process,
         levels = c("slFMD", "slQUASI", "stsl", "stst",
                    "Data from \nWilliams et al.\n(2017)"))

jpeg("/home/thom/Dropbox/R/projects/manuscripts/benchmark/3-williams/fig4.jpg",
     width = 10, height = 10, units = "in", res = 600)
library(ggplot2)
ggplot(data = fig, aes(x = AverageRecall, y = AveragePrecision)) +
  facet_grid(type ~ .) +
  geom_point(aes(color = DE, alpha = alpha, shape = Process), size = 3) +
  xlim(0, 1) + ylim(0, .5) +
  theme_bw() +
  xlab("Recall (Average Across Microarray References)") +
  ylab("Precision (Average Across Microarray References)") +
  scale_alpha_continuous(guide = FALSE) +
  labs(color = "DE Method",
       shape = "Process Method") +
  scale_colour_brewer(palette = "Set1") +
  ggtitle("Differential Expression Performance using a Microarray Reference")
dev.off()
```

```r
setwd("/home/thom/Dropbox/R/projects/manuscripts/")

###########
# funcs
###########

edgeR.williams <- function(expMat, group, method){
  library(edgeR)
  y<-DGEList(counts=expMat,group=group)
  y<-calcNormFactors(y)
  # Filter for counts present in half the samples, on cpm data
  cutoff<-ncol(expMat)/2
  keep <- rowSums(cpm(y)>1) >= cutoff
  y <- y[keep, , keep.lib.sizes=FALSE]
  ## Recalculate norm factors after filtering
  y<-calcNormFactors(y)
  y<-estimateCommonDisp(y)
  y<-estimateTagwiseDisp(y)
  et<-exactTest(y)
  adjp<-topTags(et,n=nrow(y))
  return(adjp)
}

# NOTE: differs from other ALDEx2.williams function in that effect = TRUE!!
ALDEx2.williams <- function(ct, group, method){
  ct[ct == 0] <- 1
  ct <- ceiling(as.data.frame(ct))
  if(method == "iil"){
    tt <- ALDEx2::aldex(ct, group, denom = "iqlr", test = "t", mc.samples = 128,
                        effect = TRUE)
    for(i in 1:1){
      nonDE.i <- which(rownames(ct) %in% rownames(tt[tt$wi.eBH > .05, ]))
      tt <- ALDEx2::aldex(ct, group, denom = nonDE.i, test = "t", mc.samples =
                          128, effect = TRUE)
    }
  }else if(method == "ii5"){
    tt <- ALDEx2::aldex(ct, group, denom = "iqlr", test = "t", mc.samples = 128,
                        effect = TRUE)
    for(i in 1:5){
      nonDE.i <- which(rownames(ct) %in% rownames(tt[tt$wi.eBH > .05, ]))
      tt <- ALDEx2::aldex(ct, group, denom = nonDE.i, test = "t", mc.samples =
                          128, effect = TRUE)
    }
  }else{
    tt <- ALDEx2::aldex(ct, group, denom = method, test = "t", mc.samples = 128,
                        effect = TRUE)
  }
  # Use wi.eBH column for FDR
  #ALDEx2::aldex.plot(tt, type = "MW", cutoff = .05)
  colnames(tt)[colnames(tt) == "wi.eBH"] <- "FDR"
  return(tt)
}

###########
# import
###########

ct.fmd <- read.csv("benchmark/4-rollins/ctslFMD_counts.csv",
                   row.names = 1)
ct.quasi <- read.csv("benchmark/4-rollins/ctslQUASI_counts.csv",
                     row.names = 1)
ct.stsl <- read.csv("benchmark/4-rollins/ctstsl_counts.csv",
                    row.names = 1)
ct.groups <- read.csv("benchmark/4-rollins/ct_groups.csv",
                      stringsAsFactors = FALSE)

# Remove features with ALL 0s
all0s <- rowSums(ct.fmd) == 0 |
  rowSums(ct.quasi) == 0 |
```

```
  rowSums(ct.stsl) == 0

# Check that sample names are consistent
data.frame(
  colnames(ct.fmd),
  colnames(ct.quasi),
  colnames(ct.stsl),
  ct.groups[,1]
)

###########
# DE runs
###########

library(yuck)
res := for(counts in list(ct.fmd, ct.quasi, ct.stsl))
  append(
    list(edgeR.williams(counts[!all0s,], ct.groups$caneToad.groups)),
    lapply(c("clr", "iqlr", "iil"), function(method){
      ALDEx2.williams(counts[!all0s,], ct.groups$caneToad.groups, method)
    })
  )

save(res, file = "benchmark/4-rollins/fig6-backup.RData")

###########
# overlap
###########

library(yuck)
DE := for(result in res)
  list(
    "edgeR" = rownames(result[[1]]$table)[result[[1]]$table$FDR < .05],
    "clr" = rownames(result[[2]])[result[[2]]$FDR < .05],
    "iqlr" = rownames(result[[3]])[result[[3]]$FDR < .05],
    "iil" = rownames(result[[4]])[result[[4]]$FDR < .05]
  )

library(Vennerable)
library(grid)
jpeg("benchmark/4-rollins/fig6a.jpg", width = 9, height = 3, units = "in", res =
600)
gridExtra::grid.arrange(
  grid::grid.grabExpr(
    plot(Venn(DE[[1]]), doWeights = FALSE, type = "ellipses")
  ), top=textGrob("Gene Overlap Diagram for Cane Toad Transcripts Aligned by the
slFMD Method", gp = gpar(fontsize = 16)))
dev.off()

jpeg("benchmark/4-rollins/fig6b.jpg", width = 9, height = 3, units = "in", res =
600)
gridExtra::grid.arrange(
  grid::grid.grabExpr(
    plot(Venn(DE[[2]]), doWeights = FALSE, type = "ellipses")
  ), top=textGrob("Gene Overlap Diagram for Cane Toad Transcripts Aligned by the
slQUASI Method", gp = gpar(fontsize = 16)))
dev.off()

jpeg("benchmark/4-rollins/fig6c.jpg", width = 9, height = 3, units = "in", res =
600)
gridExtra::grid.arrange(
  grid::grid.grabExpr(
    plot(Venn(DE[[3]]), doWeights = FALSE, type = "ellipses")
  ), top=textGrob("Gene Overlap Diagram for Cane Toad Transcripts Aligned by the
stsl Method", gp = gpar(fontsize = 16)))
dev.off()

# Use ImageMagick to merge
system("convert -append benchmark/4-rollins/fig6a.jpg benchmark/4-rollins/
```

```
fig6b.jpg benchmark/4-rollins/fig6c.jpg benchmark/4-rollins/fig6.jpg")

###########
# FC plot
###########

# Differences between edgeR and ALDEx2 precision not easily explained by log-fold
change
ES :- for(result in res)
  list(
    "edgeR" = result[[1]]$table$logFC[result[[1]]$table$FDR < .05],
    "clr" = result[[2]]$diff.btw[result[[2]]$FDR < .05],
    "iqlr" = result[[3]]$diff.btw[result[[3]]$FDR < .05],
    "iil" = result[[4]]$diff.btw[result[[4]]$FDR < .05]
  )

names(ES) <- c("slFMD", "slQUASI", "stsl")
ESout :- for(es in c("slFMD", "slQUASI", "stsl"))
  for(i in c("edgeR", "clr", "iqlr", "iil"))
    data.frame("process" = es, "method" = i, "FC" = ES[[es]][[i]])

library(ggplot2)
df <- do.call("rbind", ESout)
df$FC <- abs(df$FC)
g <- ggplot(df, aes(x = method, y = FC, col = method)) + facet_grid(. ~ process) +
  geom_violin() + geom_boxplot(width = .15) + scale_colour_brewer(palette = "Set1")
+
  ylab("Mean (edgeR) or Median (ALDEx2)\nBetween-Group Differences") +
  theme_bw() + xlab("Differential Expression (DE) Method") + labs(col = "DE
Method") +
  ggtitle("Absolute Between-Group Differences for DE Transcripts (Cane Toad Data)")

jpeg("benchmark/4-rollins/fig7.jpg", width = 10, height = 10, units = "in", res =
600)
plot(g)
dev.off()
```

```
setwd("/home/thom/Dropbox/R/projects/manuscripts/")

###########
# import
###########

load("benchmark/4-rollins/fig6-backup.RData")

ct.fmd <- read.csv("benchmark/4-rollins/ctslFMD_counts.csv",
                   row.names = 1)
ct.quasi <- read.csv("benchmark/4-rollins/ctslQUASI_counts.csv",
                     row.names = 1)
ct.stsl <- read.csv("benchmark/4-rollins/ctstsl_counts.csv",
                    row.names = 1)

counts <- list(ct.fmd, ct.quasi, ct.stsl)
names <- list("slFMD", "slQUASI", "stsl")

library(yuck)
DE := for(result in res)
  list(
    "edgeR" = rownames(result[[1]]$table)[result[[1]]$table$FDR < .05],
    "clr" = rownames(result[[2]])[result[[2]]$FDR < .05],
    "iqlr" = rownames(result[[3]])[result[[3]]$FDR < .05],
    "iil" = rownames(result[[4]])[result[[4]]$FDR < .05]
  )

###########
# failed recall data
###########

found.ALDEx2any := for(d in DE) union(union(d[[2]], d[[3]]), d[[4]])
found.edgeRonly := for(i in 1:length(DE)) setdiff(DE[[i]][[1]], found.ALDEx2any
[[i]])

est <- function(x) mean(x)
est.ALDEx2any := for(i in 1:length(counts))
  data.frame("name" = names[[i]], "type" = "ALDEx2",
             "value" = apply(counts[[i]][found.ALDEx2any[[i]],], 1, est))
est.edgeRonly := for(i in 1:length(counts))
  data.frame("name" = names[[i]], "type" = "edgeR only",
             "value" = apply(counts[[i]][found.edgeRonly[[i]],], 1, est))

df <- rbind(do.call(rbind, est.ALDEx2any), do.call(rbind, est.edgeRonly))
df$type <- factor(as.character(df$type), levels = c("edgeR only", "ALDEx2"))

wilcox.test(value ~ type, df)

###########
# failed recall plot
###########

base_breaks <- function(n = 10){
  function(x) {
    axisTicks(log10(range(x, na.rm = TRUE)), log = TRUE, n = n)
  }
}

jpeg("benchmark/4-rollins/fig8.jpg", width = 10, height = 10, units = "in", res =
600)
library(ggplot2)
library(scales)
ggplot(df, aes(x = type, y = value, col = type)) + facet_grid(. ~ name) +
  geom_violin() + geom_boxplot(width = .15) + scale_colour_brewer(palette = "Set1")
+
  scale_y_continuous(trans = "log10", breaks = base_breaks()) +
  ylab("Average Abundances for DE Transcripts\n(counts per sample)") +
  theme_bw() + xlab("Differential Expression (DE) Method") + labs(col = "DE
Method") +
```

```
  ggtitle("Average Abundances for DE Transcripts Called by edgeR Only (Cane Toad
Data)")
dev.off()
```
